# Novel resistance strategies to soybean cyst nematode (SCN) in wild soybean

**DOI:** 10.1038/s41598-021-86793-z

**Published:** 2021-04-12

**Authors:** Janice Kofsky, Hengyou Zhang, Bao-Hua Song

**Affiliations:** 1 Department of Biological Sciences, University of North Carolina at Charlotte, Charlotte, NC 28223 USA; 2 Present Address: Donald Danforth Plant Science Center, Saint Louis, MO 63132 USA

**Keywords:** Plant sciences, Plant stress responses, Biotic, Systems biology, Systems analysis

## Abstract

Soybean cyst nematode (SCN*, Heterodera glycine* Ichinohe) is the most damaging soybean pest worldwide and management of SCN remains challenging. The current SCN resistant soybean cultivars, mainly developed from the cultivated soybean gene pool, are losing resistance due to SCN race shifts. The domestication process and modern breeding practices of soybean cultivars often involve strong selection for desired agronomic traits, and thus, decreased genetic variation in modern cultivars, which consequently resulted in limited sources of SCN resistance. Wild soybean (*Glycine soja*) is the wild ancestor of cultivated soybean (*Glycine max*) and it’s gene pool is indisputably more diverse than *G. max*. Our aim is to identify novel resistant genetic resources from wild soybean for the development of new SCN resistant cultivars. In this study, resistance response to HG type 2.5.7 (race 5) of SCN was investigated in a newly identified SCN resistant ecotype, NRS100. To understand the resistance mechanism in this ecotype, we compared RNA seq-based transcriptomes of NRS100 with two SCN-susceptible accessions of *G. soja* and *G. max*, as well as an extensively studied SCN resistant cultivar, Peking, under both control and nematode J2-treated conditions. The proposed mechanisms of resistance in NRS100 includes the suppression of the jasmonic acid (JA) signaling pathway in order to allow for salicylic acid (SA) signaling-activated resistance response and polyamine synthesis to promote structural integrity of root cell walls. Our study identifies a set of novel candidate genes and associated pathways involved in SCN resistance and the finding provides insight into the mechanism of SCN resistance in wild soybean, advancing the understanding of resistance and the use of wild soybean-sourced resistance for soybean improvement.

## Introduction

The soybean (*Glycine max* (L.) Merr.), is an important legume crop that supplies more than half of the word’s vegetable fats, oils, and protein meal^1^. Soybean cyst nematode (SCN *Heterodera glycines* Ichinohe) is the most destructive soybean pest^2^, and the distribution of SCN has spread rapidly since the initial detection in 1954 in North Carolina, due to natural spread of pathogens and the lack of resistant cultivars^3^.

Development of SCN resistant soybean cultivars in the United States are dependent on few sources of resistance. There are three sources of SCN resistant *G. max* primarily used for breeding commercial varieties in the United States: PI88788, Peking (PI548402), and PI437654. The majority, over 95%, of which are sourced from PI88788^4^. In 2005, 94% of the SCN resistant cultivars grown in Illinois, sourced from PI88788, were no longer resistant to the majority of SCN populations found in the soil, including SCN race 5^5^. In fact, SCN resistant cultivar, Peking, is the main source of resistance to race 5 in the United States, and only makes up less than 5% of the cultivars used. The least used cultivar, PI437654, demonstrates broad resistance and is able to resist race 5, although it is rarely used in breeding practice due to complications with the genetic background and linkage to less favorable traits^6^. Nematodes that are adapted to PI437654-type resistance are able to overcome all other sources of resistance, and therefore cultivars sourced from this accession should be used sparingly^7,8^. Nematodes have demonstrated the ability to adapt and overcome all resistance found in the *G. max* gene pool^5,7–9^. Thus, there is a high demand for new sources of SCN resistance.

Research on SCN resistance dates back to 1960, with the discovery of the first *rhg* loci (resistant to *Heterodera glycines*)^10^. It was initially assumed that the mechanism of SCN resistance in the cultivated soybean was comprised of LRR-Kinase genes, which mapped closely to *rhg* loci and were known to confer resistance in other crop species^11,12^. It was later found that resistance by the two loci with larger effects, *rhg1* and *Rhg4*, was independent of LRR-Kinase genes^13,14^, and instead is conferred by a novel resistance strategy. A complex mechanism of resistance in the cultivated soybean consists of copy number variation of causative genes at the *rhg1* and *Rhg4* loci, and the genetic composition of these genes and associated promoters^15–20^. Two main types of resistance are found within the cultivated soybean, Peking-type and PI88788-type, with Peking-type being the only type resistant to SCN race 5(4). Peking-type resistance is conferred by low-copy *SNAP* (soluble NSF attachment protein) gene (*Glyma.18G022500,* alternative ID: *Glyma18g02590*) at *rhg1-a* in combination with resistant-type *SHMT* (serine hydroxymethyltransferase) gene (*Glyma.08G108900*, alternative ID: *Glyma08g11490*) at *Rhg4*^18,21^. Broad resistance in PI437654 is derived from Peking-type, with a high-copy *SHMT* allele^20^. PI88788 type resistance is conferred by a high-copy number of three genes, including *SNAP*, at rhg1-b, with no presence of resistant-type *Rhg4*^15,16^. Duplication and selection at resistant *rhg1* occurred in wild soybean, but the resistant *Rhg4* allele emerged after domestication^22,23^. It is likely that the wild soybean population has ancestral and separately derived strategies to combat SCN, not found in the cultivated gene pool.

The wild counterpart to the cultivated soybean, *G. soja* (Siebold & Zucc.), is analyzed for SCN resistance novel to the crop gene pool. The domestication bottleneck resulted in an 81% loss of rare alleles, 60% gene allele frequency change, and nucleotide diversity (π) was almost halved in cultivated soybean relative to wild soybean^24^. After domestication, selection toward the elite cultivars resulted in an additional loss of 23% (π) nucleotide diversity, and a 21% loss of rare alleles^24^. Due to the dilution of the cultivated gene pool, half of the resistance-related sequences in *G. soja* are not found in landraces or the domesticated soybean^25^. Selection within *G. max* alone has been shown to have a significant effect on diversity across the entire genome^26^. SCN is likely native to China and still prevalent throughout the native range of wild soybean^27,28^. Genetic isolation of environmental niches suggests that there is strong selection of environmentally tailored adaptations, such as nematode resistance, within the wild soybean gene pool^29,30^. Novel traits such as pest and disease resistance from wild relatives have been incorporated into major crops^31–40^, but progress of this sort is lacking in soybeans^30,41–43^. *G. soja* has been shown to exhibit native resistance to SCN populations currently inflicting the United States crops^5,44^, and only a few studies have reported candidate genes for SCN resistance in *G. soja*^5,44,45^. Further research on *G. soja* ecotype, S54, has revealed a mechanism to confer resistance that is not related to the currently known mechanism in *G. max*^45–47^. Since wild soybean retains rare alleles that have been lost in cultivated soybean^24^, SCN resistance identified in wild soybean is expected to be novel, and can be used to mitigate SCN damage and rapid spread in soybean cultivars. SCN resistance is complex, and additional understanding of the mechanism in wild soybean is needed to benefit SCN resistance improvement.

In the present study, we dissected the mechanism of resistance found in wild soybean ecotype, NRS100 (nematode resistant *soja,* PI578345). This ecotype is significantly resistant (Female Index = 3.3) to SCN HG Type 2.5.7 (SCN race 5), which is prevalent in the south-eastern United States. To reveal the mechanism of resistance, we compared NRS100 SCN response with SCN race 5-resistant cultivar, Peking. A direct comparison between resistance in wild *G. soja* and cultivated *G. max* has not been reported previously. Here, we compared RNA-seq transcriptomes of SCN treated and control conditions across four genotypes: Peking (PI548402), Williams 82 (PI509044), susceptible *soja* (PI468396B, S-*soja* hereafter*)*, and NRS100 to determine differential regulation between genotypes in response to race 5. Combined analysis of nematode susceptible and resistant genotypes from both cultivated and wild soybeans allows us to elucidate species-specific and genotype-specific responses to race 5. Novel resistance mechanisms of resistance were found within NRS100, which will further facilitate an understanding of resistance mechanism, and future development of new SCN resistant soybean cultivars.

## Results

### NRS100 is highly resistant to SCN race 5

Our previous study showed striking variation in resistance levels to SCN race 5 after screening a set of 235 wild soybean accessions^44,45^. A bar plot was used to visualize of the resistance level of the 235 genotypes studied, indicating NRS100 and S-*soja* specifically (Fig. S1). NRS100 showed high resistance to SCN race 5 with a Female Index (FI) of 3.3 and S-soja is highly susceptible with a FI of 149^44,45^. The validation screening result confirmed the high resistance of NRS100 to race 5.

### The expression patterns of *rhg1* and *Rhg4* in NRS100

**Figure 1:**
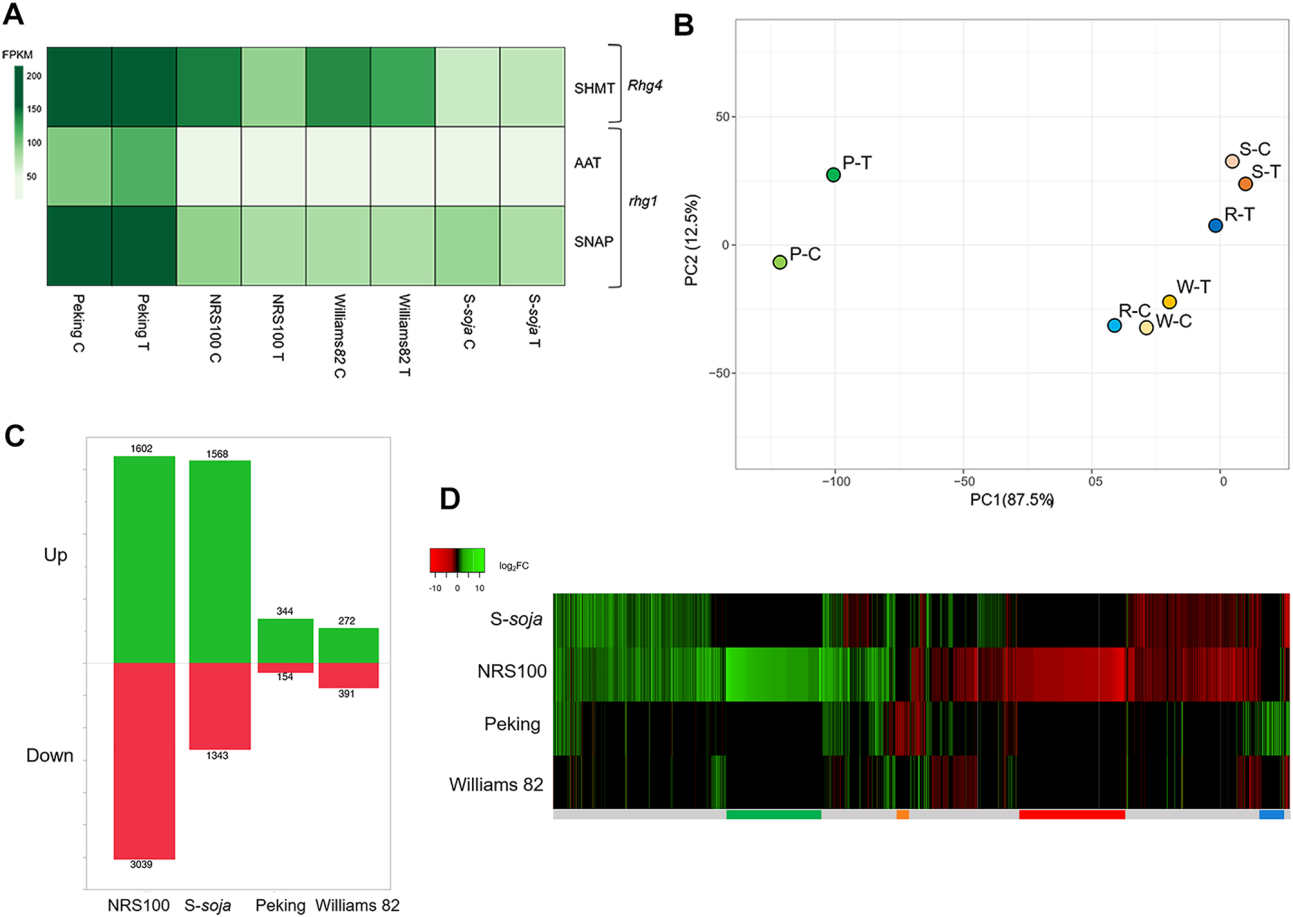
(**A**) Relative expression of *rhg1* genes AAT (*Glyma.18G022400*) and SNAP (*Glyma.18G022500*), and *Rhg4* gene *SHMT* (*Glyma.08G108900*). No clustering, scaling, or imputed values. Sequential color scheme shows FPKM values. (**B**) Probabilistic principal component analysis (PCA) plot of expression of genes *SNAP* (*Glyma.18G022500*) and *SHMT* (*Glyma.08G108900*). Individual points represent FPKM of treatment groups P–C (Peking control), P–T (Peking SCN treated), R–C (NRS100 control), R–T (NRS100 SCN treated), W–C (Williams 82 control), W–T (Williams 82 SCN treated), S–C (S-*soja* control), S–T (S-*soja* SCN treated). (**C**) Significantly up and down regulated genes by genotype. DEGs considered significant if q value < 0.01, log_2_foldchange no less than ± 0.6. (**D**) Heatmap of all DEGs by log_2_FC (fold change) of induced response to SCN treatment of all genotypes. Cutoff Log_2_FC no less than ± 2 for NRS100, including 2076 genes. Green bar indicates NRS100 specific up regulated genes in response to SCN stress. Red bar indicates NRS100 specific down regulated genes in response to SCN stress. Blue bar indicates Peking specific up regulated genes in response to SCN stress. Orange bar indicates Peking specific down regulated genes in response to SCN stress.

We analyzed the expression patterns of *rhg1* and *Rhg4*, which are the well-studied loci conferring SCN resistance in cultivated soybean PI88788 and Peking^17,19,48,49^. The *rhg1* locus differs between Peking-type (*rhg1-a*) and PI88788-type (*rhg1-b*), but shares the AAT (amino acid transporter) gene (*Glyma.18G022400*, alternative ID: *Glyma18g02580*) and SNAP gene. Peking-type resistance is conferred by SNAP gene at *rhg1* and *SHMT* at *Rhg4* each, and respectively^18,20^. Relative FPKM (Fragments Per Kilobase of transcript per Million mapped reads) of SHMT at *Rhg4*, AAT at *rhg1,* and SNAP at *rhg1* for all four studied genotypes at control and SCN treatment conditions revealed different expression profiles (Fig. 1). Expression of *rhg1*-b genes in PI88788-type resistance was not significant in NRS100. A principal component analysis of expression of SHMT and SNAP, confirmed distinct expression profiles (Fig. 1B). Noticeably, NRS100 did not exhibit any significant difference in expression at *rhg1* or *Rhg4* from the SCN susceptible wild soybean, S-*soja*, or cultivar, Williams 82.

Analysis of the transcript sequence of SNAP at *rhg1* and SHMT at *Rhg4* revealed Peking specific variations not found in the other three genotypes. In SNAP, Peking has two non-synonymous SNPs, one at 1,634,660 bp on exon 6 (G/C), resulting in an amino acid change from Aspartic acid to Glutamic acid. The other at 1,645,409 bp on exon 9 (A/C), resulting in an amino acid change from Threonine to Asparagine (Fig. S2). In addition, a *G. soja*-specific synonymous variation exists on at 1,641,767 bp at exon 2 (G/A). Another synonymous variation exists at 1,641,789 on exon 2, specific to NRS100 (G/A) (Fig. S2). The only non-synonymous variations in SNAP are found in Peking, while NRS100 has a similar susceptible-type SNAP gene similar to Williams 82. A similar trend was seen in the SHMT transcript sequence. A synonymous SNP was found in NRS100 at 8,361,269 bp (C/T) (Fig. S3). A non-synonymous variation was found in Peking at 8,361,924 bp (A/T), resulting in an amino acid change from Asparagine to Tyrosine (Fig. S3), as well as distinct variation in mapping from the reference (Williams 82) in this region, which was not found in wild soybean NRS100 or S-*soja*. All sequence variations that resulted in amino acid changes were found only in Peking-type SNAP and SHMT sequences.

The resistance conferring gene of *rhg1-a* in Peking-type resistance, SNAP gene, is located at chromosome 18. Paralogous SNAP genes were analyzed to determine if resistance in NRS100 was conferred by different members of the SNAP gene family. No significant expression or fold change is found on SNAP 2, 9, 11, or 14 by RT-qPCR or RNA-sequencing, as if found in resistant soybean cultivars using SNAP for resistance^50^. The results showed that fold changes for all NRS100 genes < 0.4 between SCN treatment vs control (Fig. S4), which suggested that NRS100 does not have significant differential expression at SHMT, SNAP 18 or a SNAP paralog.

### Expression patterns of previously identified candidate genes involved in SCN resistance in wild soybean

Two major QTLs, *cqSCN-006* on chromosome 15 and *cqSCN-007* on chromosome 18, have been previously identified involved in SCN resistance to race 5 in wild soybean genotype PI468916^51–53^. Interestingly, none of the candidate genes on *cqSCN-006* were found significantly upregulated in NRS100, including the *y*-SNAP gene (*Glyma.15g191200*), while one candidate gene, Apetala 2 (*AP2*), on *cqSCN-007* was significantly upregulated in NRS100. This region also collocates with SCN resistance loci qSCN18 in soybean landrace PI 567516C^54^. *AP2* is a, transcription factor (*Glyma.18g244600*)^53^, known to regulate multiple developmental pathways and play a role in abiotic and biotic stress response^55^.

A previous RNA-sequencing study of a SCN race 5 resistant wild soybean genotype S54, revealed potential pathways involved in resistance in this genotype^46,47^. Expression patterns of S54 and NRS100 were compared to determine if the mechanism of resistance was the same. Many differences between NRS100 and S54 were found, similarities between these genotypes were limited to the following. Previously identified Leucine-rich repeat receptor-like protein kinases (LRR-RLKs) differentially expressed genes (DEGs) in *G. soja* s54 SCN response, Brassinosteroid insensitive 1 (BRI1)-associated kinases (BAK1) (*Glyma.05G119500*, *Glyma.05G119600*) genes and BAK-1interacting receptor kinase 1 (SOB1R1) genes *(Glyma.04G190400, Glyma.06G175100)* were also induced in response to SCN in NRS100, but not exclusively to this genotype^46^. Two genes (*Glyma.02G270700, Glyma.15G111300*) of chitin elicitor receptor kinase 1 (CERK1) were down and upregulated respectively in NRS100, a similar patten was seen in S54 SCN race 5 response. Lectin receptor kinase (*Glyma.07G135400*) was also induced in NRS100, which was also found in previously studied S54^46^. In addition, significant downregulation of NBS-LRR gene (*Glyma.16G209000*) and upregulation of NBS-LRR gene (*Glyma.17G180000*) was found in NRS100, like in S54. Significant upregulated DEGs associated with calmodulin binding (*Glyma.05G237200*, *Glyma.07G093900*, *Glyma.08G044400*) were upregulated in NRS100 and S54. Calcium transport genes: autoinhibited Ca2+-ATPase 9 (ACA9, Glyma.07G004300) and protein calcium exchanger 7 (CAX7, Glyma.19G066700) were upregulated in both NRS100 and S54. MAPKKKs (*Glyma.17G245300* and *Glyma.05G094400*) were significantly up and down regulated in both S54 and NRS100, however, the strongest DEG in the MAPK cascade found in S54 (*Glyma.15G048500*), was not found to exhibit the same response in NRS100. Jasmonic acid (JA) pathway gene, oxophytodienoate-reductase 3 (DDE1/OPR3, *Glyma.13G109800)* showed a similar induced effect in NRS100 as in S54, however this was the only one gene of the JA pathway genes to show this similarity between the two genotypes. Members of WRKY40 transcription factor (*Glyma.13G370100* and *Glyma.*17G222500) were found highly induced in NRS100 and S54, although much higher in S54. No similarities were found in the upregulated chitinase genes in S54, however, members of Chitinase-like protein (CLT), *Glyma.09G038500* and *Glyma.15G143600*, were found down regulated in both NRS100 and S54^46^. Overall, similarities between expression in NRS100 and S54 are limited, suggesting a different regulatory mechanism of resistance between NRS100 and S54.

### Induced gene expression in response to SCN infection in NRS100

The entire RNA-seq based transcriptome comparison of NRS100 with the other genotypes, including susceptible wild soybean genotype S-*soja*, as well as resistant cultivar Peking and susceptible cultivar William82, was conducted to understand the unique resistance mechanism in NRS100. Illumina sequencing generated 17.9–29.7 million raw reads per library. Filtering by quality of reads resulted in 93.8–95% high quality (quality score > 20) reads for alignment. The mapping rate of quality-controlled reads ranged from 84.9 to 92.8% of all 24 libraries to *G. max* Wm82.a2.v1 (Table S1). On average, 56, 710 expressed genes were found in root tissue from each genotype. The amount of significantly (q < 0.01) differentially expressed genes between control and treatment groups for each genotype were 4641, 2911, 498, and 663 in NRS100, S-*soja*, Peking, and Williams 82, respectively.

To determine the genes associated with induced resistance response to SCN in NRS100, DEGs of four genotypes between control and SCN treatment were compared. A count of the significant DEGs of all four genotypes, NRS100, S-*soja*, Peking, Williams 82, in response to SCN infection revealed a greater quantity of expressed genes in NRS100 compared to other genotypes, with 1602 and 3039 genes significantly up and down regulated respectively (Fig. 1C). The SCN susceptible wild soybean (S-*soja*) has the second most substantial response to SCN infection. Both cultivars, Peking and William 82, exhibit less extensive and more tailored response to SCN infection, with Peking exhibiting a lower number of down regulated genes. This trend is further supported by the expression profiles of highly-significantly expressed genes, with a cutoff Log_2_FC no less than ± 2 for NRS100 (Fig. 1D).

NRS100 is seen to have a different set of genes induced in response to SCN infection, (green and red bars (Fig. 1D), when compared to other three genotypes. By contrast, the induced response to SCN infection in Peking (blue and orange bars (Fig. 1D), is controlled by a smaller set of genes, separate from those induced in NRS100. Both shared responses by all genotypes, and species-specific shared responses were seen. GO enrichment analysis of resistance specific, shared response, and stress response genes was done after visualization of all DEG’s in a Venn-diagram^56^ (Fig. S5).

**Figure 2:**
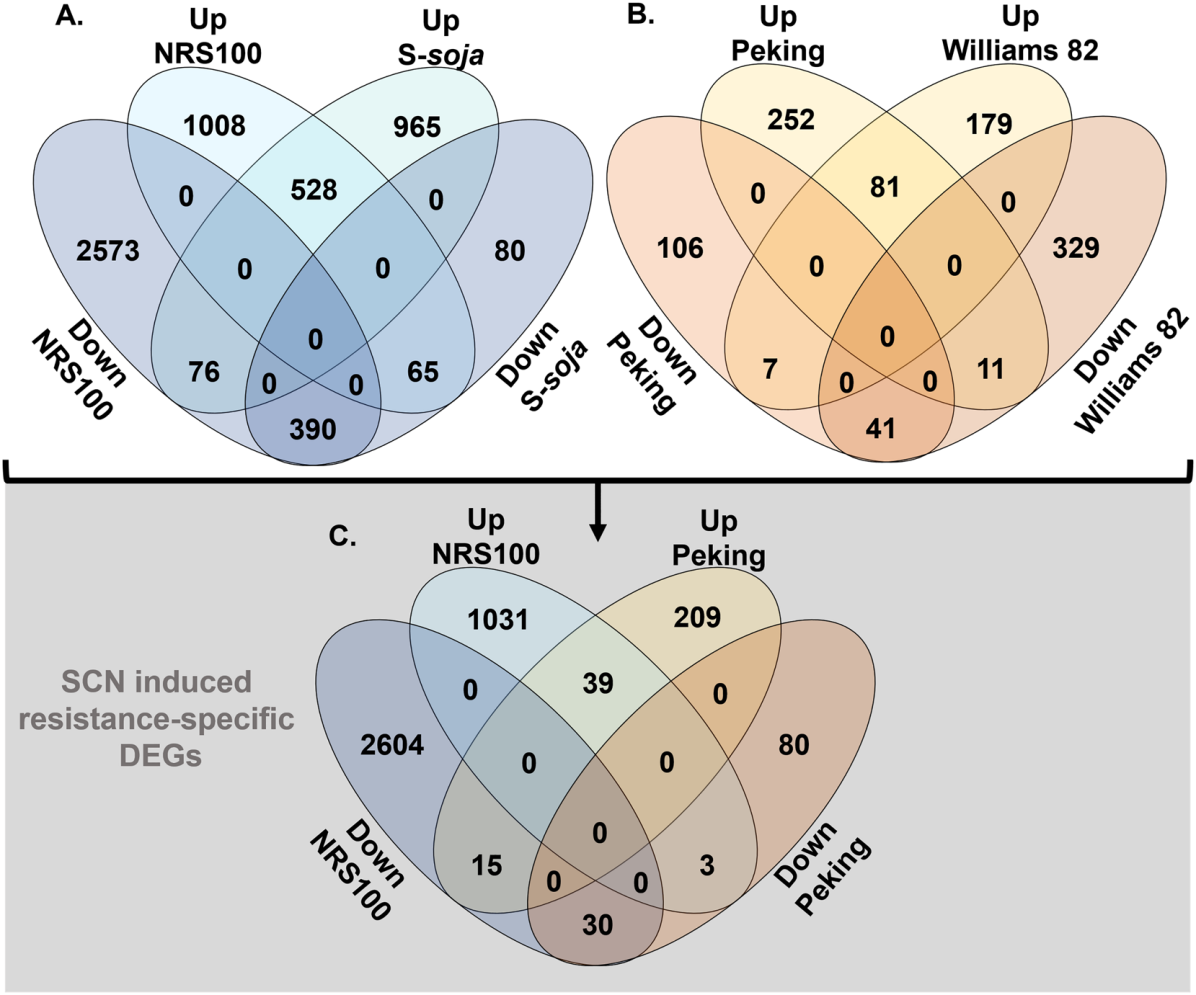
(**A**) Comparison of DEGs for SCN susceptible and SCN resistant *G. soja* genotypes. (**B**) Comparison of DEGs for SCN susceptible and SCN resistant *G. max* genotypes. Shared responses from susceptible and resistant genotypes from the same species were eliminated to produce a comparison of resistance-specific associated genes. (**C**) Comparison of resistance-specific genes between Peking and NRS100.

To elucidate the expression profile associated with resistance and eliminate generalized stress responses, species-specific induced responses were compared (Fig. 2A,B). Shared induced responses by genotype were eliminated to create a subset of resistance-specific induced DEGs (Fig. 2C). Again, resistance-specific expression in NRS100 is at a much larger scale than that in Peking, consisting of expression profile more than ten-fold to Peking. Comparison of resistance-specific expression profiles of Peking and NRS100 reveal a shared response consisting of 69 genes, contributing to only 1.8% of the overall resistance-specific profile of NRS100. GO functions associated with NRS100 resistance include up regulation of systemic acquired resistance mechanisms, salicylic acid signaling pathway, and regulation of defense response mechanisms (Fig. S5, Tables S2, S3) and down regulation of secondary cell wall biogenesis and light harvesting by photosynthesis (Tables S3, S4).

### Constitutive gene expression in NRS100

To further analyze the potential mechanism of SCN resistance in NRS100, the constitutive expression profile specific to NRS100 was considered due to its important role in plant-pathogen protection^57–61^. To determine the constitutively expressed genes associated with SCN resistance in NRS100, the basal regulation of NRS100 was compared to S-*soja* and Peking at control conditions. This eliminates specific-specific regulation shared with wild soybeans and resistance-specific regulation shared with cultivars. The significantly induced expression profile from SCN infection in NRS100 was also eliminated from the basally regulated genes. This method ensures that only the genes highly expressed in the control condition in NRS100 specifically, that also did not significantly alter their expression after SCN infection, were captured. Comparison of all expression profiles results in a set of 1656 genes that are constitutively expressed in NRS100, (Fig. S6). The overrepresented GO functions of these genes include pathways associated with photoprotection, defense response, and respiratory burst defense response, indicative presence of polyamine derived hydrogen peroxide (Fig. S7)^62,63^. Analysis of combined induced and constitutive NRS100 specific upregulation revealed the importance of glutamine metabolic process (GO:0006541), glutathione metabolic process (GO:0006749), and chitin binding (GO:0008061) molecular functions. Associated biological processes include defense and stress response processes (GO:0006950, GO:0006952, GO:0006979) and response to biotic stimulus (GO:0009607, GO:0043207). The cellular component of these processes was allocated to areas including the cell periphery (GO:0071944), plasma membrane (GO:0005886), extracellular region (GO:0005576), and cell wall (GO:0009505) (Table S5).

### Expression patterns in NRS100-specific SCN resistance pathways

Based on the above findings, putative metabolic pathways conferring resistance were selected based on exclusive constitutive and induced responses, importance in GO enrichment, and previous understanding of plant–pest interactions. The jasmonic acid (JA) signaling pathway is a well-studied pathway, associated with plant-pathogen interactions, and known to have antagonistic effects on the salicylic acid (SA) signaling pathway, and therefore was analyzed further to determine if signal transduction plays a role in SCN resistance in NRS100^64–68^. Within the JA signaling pathway, NRS100-specific downregulation occurred at JAR1 (jasmonate resistant 1) genes and COI1 (coronatine-insensitive protein 1) gene. JAR1 genes, *Glyma.03G256200* and *Glyma.16G026900*, were exclusively down regulated in NRS100 in response to SCN. Downstream of JAR1, COI1 gene, *Glyma.18G030200*, was downregulated in NRS100 exclusively in response to nematodes. No other genotypes had a significant induced response at these genes. Regulation at known genes in JAZ (jasmonate ZIM domain-containing protein) and MYC2 transcription factor (*Glyma.08G271900*) were not induced in NRS100 but were highly expressed in control conditions, or constitutively expressed significantly higher than other genotypes (Fig. S8). Expression at these significant genes in the JA signaling pathway were confirmed in the RT-qPCR results with representatives for JAR1 and JAZ due to modulation of these proteins by multiple genes (Table S6).

The polyamine biosynthesis pathway, specifically at spermidine synthesis, was analyzed in further detail to determine if NRS100 uses a polyamine related defense response known to play a role in resistance to other plant pathogens^69,70^. Upstream of spermidine synthase, ODC1 (ornithine decarboxylase, *Glyma.04G020200*) is significantly upregulated in response to nematode infection in both Peking and NRS100. There are two paralogous spermidine synthase genes, SPDS17 (*Glyma.17G091123*) and SPDS5 (*Glyma.05G036300*). SPDS5 is significantly upregulated in both NRS100 and Peking. However, SPDS17 is significantly upregulated in response to nematode infection in NRS100 only. These expression results are confirmed by both RNA-sequencing and RT-qPCR results. Downstream of spermidine synthesis, expression of PR-1 (pathogenesis-related protein 1) genes is highly constitutively expressed in NRS100 at *Glyma.15G062300*, and non-specifically significantly induced at *Glyma.15G062400* and *Glyma.15G062500*. All three PR-1 genes are significantly induced in response to SCN infection in S-*soja*, but not in either cultivar. Species-specific response at PR-1 is found, with NRS100 specific constitutive expression of *Glyma.15G062300 and* overall higher level of expression when induced in NRS100. The polyamine related defense response includes direct and indirect resistance mechanisms, where indirect involves the production of an unknown secondary metabolite.

### Validation of expression by RT-qPCR

**Figure 3:**
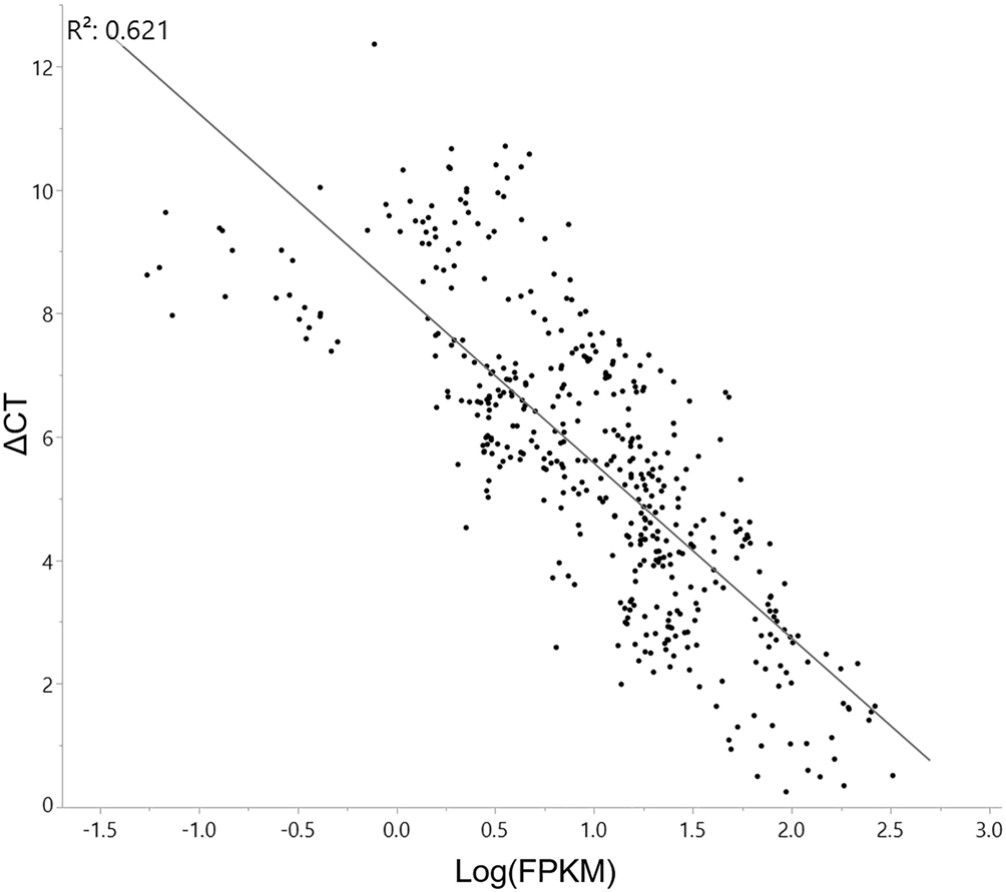
Correlation (R^2^ = 0.621) between qPCR expression values (ΔCT) and correlated RNA-sequencing expression values (FPKM) for 20 genes. Individual points represent relative expression of individual replicates calculated by RT-qPCR analysis 2^−ΔΔ CT^ method and calculated FPKM expression of individual replicates from RNA-sequencing analysis.

RNA-sequencing expression of 20 genes (Table S6), including randomly selected and selected from important pathways detailed in PATHVIEW results (Fig. S8), was validated using RT-qPCR for all genotypes and condition replicates. RT-qPCR results of the 20 selected genes significantly correlated with RNA-sequencing results. An expected indirect correlation (R = 0.621) of RT-qPCR derived ΔCT to RNA-sequencing derived FPKM of all biological replicates and averaged technical replicates was found (Fig. 3). In addition, fold changes found in RT-qPCR significantly correlates (R = 0.762) with that from RNA-sequencing data (Fig. S9). RT-qPCR was also used to better understand the expression of genes of interest, including those within *rhg1*, *Rhg4*, SNAP paralogs, the JA signaling pathway, and the Polyamine defense response.

## Discussion

SCN is the most destructive pest to soybean, and is rapidly evolving enhanced and complex virulence against resistant crops^2,4,9,71^. The majority of efforts to understand SCN resistance mechanisms is focused on PI88788 and Peking type resistance, which use *rhg1-b* or a combination of *rhg1-a* and *Rhg4*^15–20,72^. Little effort has been placed on sources of resistance loci outside *rhg1* or *Rhg4*, but is necessary to combat current and evolving SCN populations. Due to the long history of environmental adaptation in natural populations, as well as the fact that the resistant *Rhg4* allele emerged after domestication, it is likely that the wild soybean has different strategies to battle SCN stress^22,23^. However, research to understand the mechanisms of resistance in wild *G*. *soja* is lacking, and only a handful of studies describe potential mechanisms^44–47,51–53,73,74^.

### The molecular mechanisms found in cultivated soybean cultivars could not explain NRS100 resistance

Our results showed that NRS100 uses a resistance mechanism independent of Peking-type or PI88788-type resistance: first, the genes at *rhg1-b*, *rhg1-a*, and *Rhg4*, were not induced in response to SCN infection in NRS100 (Fig. 1A). Second, analysis of the molecular sequence of exons in SNAP and SHMT revealed that non-synonymous mutations found in Peking were not found in any other genotypes in our study, including NRS100. This confirmed that the Peking-type variation of these resistance genes were not found in NRS100. It is known that resistance to SCN is conferred by a complex mechanism in the cultivated soybean, which consists of variation in copy number and genetic composition of genes at *rhg1* and *Rhg4* and associated promoters^15–20^. Our results suggest NRS100 does not make use of *rhg1* and *Rhg4* based on transcription of these genes constitutively and induced, and molecular sequence of the coding region, but copy number of these genes and composition of their promotors are yet to be determined.

It has been suggested that the resistance mechanism involvig SNAP in Peking is likely due to interactions of the SNAP product with proteins within the SCN secretion at the point of infection, such as Hg-SLP-1^72,75,76^. Due to the importance of SNAP in SCN resistance, the SNAP family was analyzed further to determine if another member can help confer resistance by a similar mechanism. Previous characterization of the SNAP family revealed that SNAP 11 contributes to additive resistance to SCN in Peking-type resistance, however our results suggest that none of the SNAP members in the family (SNAP2, SNAP9, SNAP11, SNAP14, and r*hg1* SNAP18) are significantly highly expressed during SCN infection in NRS100. In addition, NSF gene, *Glyma.07g195900*, previously shown to be associated with resistant SNAP at *rhg1,* is not significantly induced or highly constitutively expressed in NRS100^50,72^. Thus, we suggest that a novel mechanism, different from the previously identified *rhg1*, *Rhg4*, and SNAP family, confer resistance in wild soybean NRS100.

### Genotype-specific SCN resistance in wild soybean

One of the most well studied wild soybean *G. soja* genotype for SCN resistance, thus far, is PI468916. The fine mapping studies revealed two candidate loci, *cqSCN-006* on chromosome 15 and *cqSCN-007* on chromosome 18^51–53^ are the two major QTLs conferring SCN resistance to race 5. None of the candidate genes on locus *cqSCN-006* were found significantly upregulated in NRS100, including the *y*-SNAP gene (*Glyma.15g191200*). However, one candidate gene, Apetala 2 (AP2), on locus *cqSCN-007* was found significantly upregulated in NRS100. AP2 is a transcription factor that is related to flower development and stress response in Arabidopsis (*Arabidopsis thaliana*)^53,55^. AP2 can be activated by either JA or SA pathways, which are established resistance-critical pathways^77–80^. It is plausible that *cqSCN-007* might play a role in SCN resistance in NRS100 at AP2 specifically, however differences in regulation of other candidate genes between PI468916 and NRS100 indicates a genotype-specific SCN resistance mechanism in each ecotype.

Another well studied wild soybean genotype for SCN resistance is S54^44^. Similar responses to SCN race 5 in NRS100 and S54 were limited to RLK-LRRs, Non-LRR domain RLKs, calmodulin binding genes, calcium transport genes, WRKY40, a JA pathway associated gene, MAPKKKs, and downregulated CLTs^44^. Moreover, the expression profile found in NRS100 did not include the important genes involved in the proposed resistance mechanism in S54^46^, which indicates that NRS100 might use a different strategy to confer SCN resistance to race 5. It is not surprising that genotype-specific resistance is found in wild soybean, since the two well-studied soybean cultivars, Peking and PI88788, also have genotype-specific resistance mechanisms to SCN.

### Novel mechanism of SCN resistance in NRS100

NRS100 displays a strikingly different expression profile with and without SCN inoculation, compared to SCN resistant soybean cultivar Peking, indicating different mechanisms employed in resistance. The amount of DEGs in response to SCN is much greater in NRS100, with twice as many DEGs than the response of susceptible *S-soja*, and almost tenfold the amount found in Peking or Williams 82. This strong response in NRS100 suggests that resistance might be more complex, and less tailored than that found in the cultivated Peking-type resistance. NRS100 and Peking have a limited number of shared responsive genes to SCN, consisting of 39 up- and 30 down-regulated genes, which is much smaller amount compared to the 1031 up- and 2604 down-regulated genes specific to NRS100. There might be a regulatory network specific to NRS100 that is non-existent in the SCN resistant cultivar, Peking, or either susceptible genotype, which has not been directly demonstrated in any other *G. soja* ecotype.

In addition to the clearly induced effect, constitutively expressed genes, or genes that are highly upregulated in control conditions of NRS100 compared to the other genotypes, suggest a resistance mechanism that is already turned on without SCN infection. Constitutively expressed genes are known to play a significant role in plant–pathogen resistance, which can promote toxin production, increase the sensitivity to pathogen stimuli, or support an inducible immunity^57–61^. Intracellular signal transduction is highly upregulated in the control NRS100 roots, suggesting a higher sensitivity to pathogen trigger via signal transducers. Ethylene biosynthesis, another signaling molecule, is also highly expressed, which was reported associated with induced resistance to root knot nematode and SCN, but conversely found to increase susceptibility to SCN in a separate study^81–86^. To explain the unique resistance found in NRS100, we hypothesized two modes of resistance in NRS100, (1) induced immunity by suppression of JA signaling pathway, and (2) spermidine synthesis mediated induced defense against SCN growth.

### Jasmonate acids (*JA*) suppression in induced immunity

Signaling pathways are widely studied for their role in pest resistance in plants. Signaling pathways, including JA, SA, and ethylene act as detection system to decipher pest and pathogen signals in order to allow a plant to mount a defense^68,87^. High levels of jasmonate acids (JA) are associated with increase wound signaling to insect pests by inducing an effective defense against pests, and induced systemic defense in root knot nematode by PR1^65,85,88,89^. However, JA signaling is antagonistic with SA signaling^87,90^. It has been shown in tomato (*Solanum lycopersicum)* that JA signaling actually has a negative impact on resistance to aphids and root knot nematode, and JA-deficient mutants exhibit elevated resistance^91,92^. In the interaction between tomato and aphids, susceptibility can be restored by suppressing the SA signaling pathway, demonstrating the direct interplay between the JA and SA signaling pathways^90^. A separate example exists in rice where modification of JA signaling increases SA levels and in turn leads to increased resistance to multiple insect herbivory^93^. It is likely that JA is important for broad resistance and wounding response, but can be costly due to its negative interaction with SA signaling which is needed for constitutive and inducible resistance mechanisms^67,94–96^. In addition, suppression of the JA signaling pathway can promote plant growth via activation of PIF regulated gibberellin signaling^97^. It is also possible that JA signaling is suppressed by a deactivation of NRS100 defense mechanisms by a decoy produced by the nematode, similar to that found in the tobacco-*Helicoverpa zea* larvae interaction^67,98^. However, this is unlikely considering this suppression is only found in NRS100 and not the SCN susceptible genotypes. With suppression of JA, SA can accumulate. SA accumulation has been found to be important in specialized defense response in Arabidopsis^99^.

**Figure 4:**
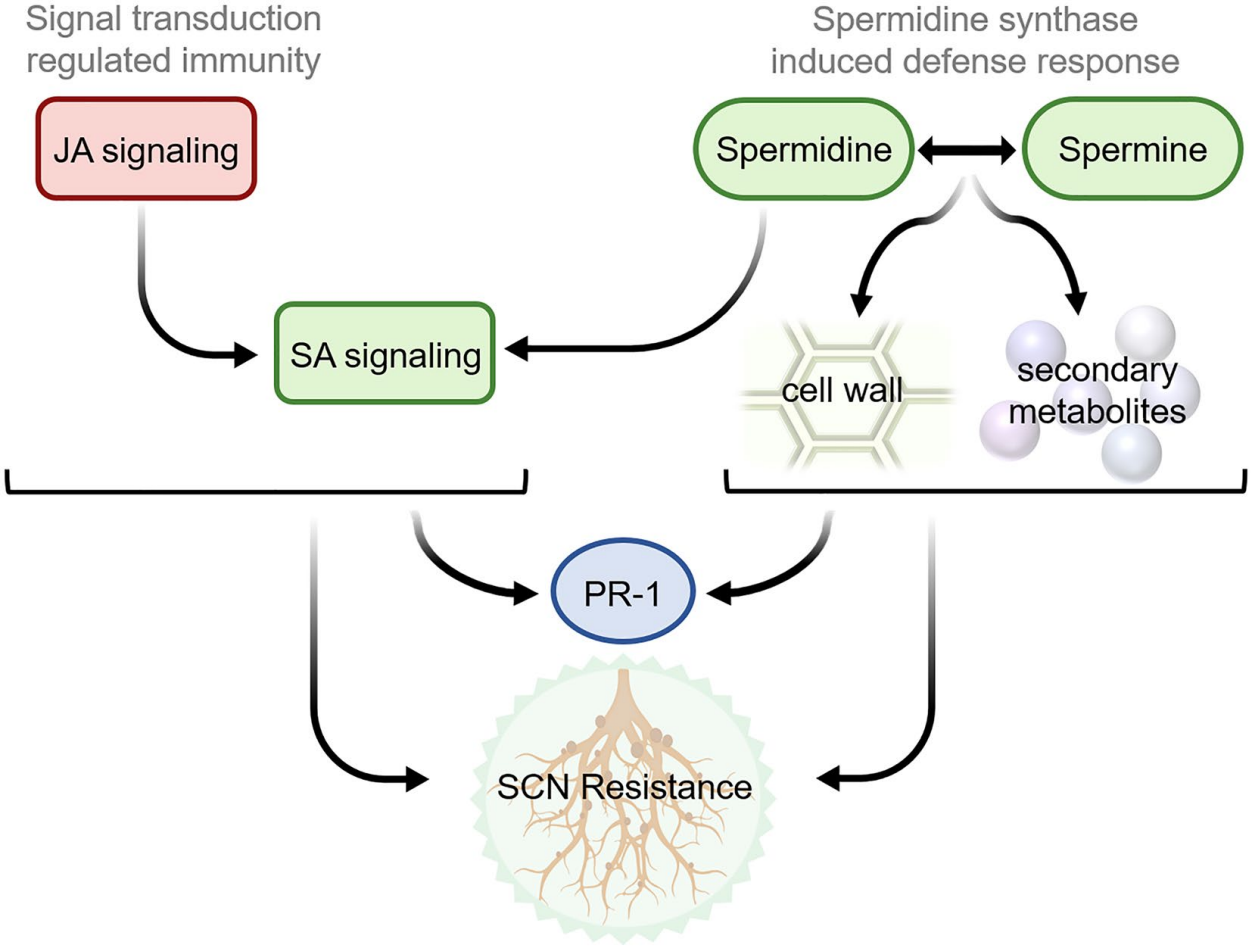
Pathway of resistance in NRS100 includes upregulation of SA signaling by JA suppression, induced PR-1 genes, accumulation of polayamines (spermidine and spermine), reinforcement of the cell wall, and production of secondary metabolites.

In NRS100, JA signaling is suppressed at JAR1 and COI1, important regulators in the pathway^89,100,101^. This suppression reduces the ability of COI1 and JAZ to interact physically, and does not allow a release of JA mediated transcription factors^89^. Upregulation of JAZ and MYC2 act as repressors to the JA pathway^102,103^. Suppression of JA allows SA signaling to induce a defense response, avoiding potential conflicts with SCN-produced decoys to mediate traditional JA signaling in SCN defense response (Fig. 4). Interestingly, the downregulation of JAR1 and COI1 is induced in NRS100 only, but the induced response at JAZ is found in both Peking and NRS100 but regulated by separate genes. This suggests that JA signaling is not only regulated differently at key steps (COI1 and JAR1) in NRS100 but is regulated by different genes at points of similar expression of with Peking (JAZ). It is likely that many different JAZ products exist, especially in the case of regulation by separate genes, which is expected to occur in NRS100^67,96^.

### Spermidine synthesis mediated induced defense against SCN growth

We hypothesize that spermidine synthesis and secondary metabolite production induces defense against SCN growth and syncytium formation in NRS100. Polyamine metabolism, including spermidine synthesis, is often altered in abiotic and biotic stress responses in plants^104,105^. Spermidine synthesis upregulation can result in accumulation of free spermidine or lead to the production of polyamine derived molecules. This can trigger H_2_O_2_ regulated hypersensitive response, cooperative effects with SA signaling to induce PR proteins or other modes of pathogen resistance, lead to production of secondary metabolites like nicotine, and reinforce/maintain structural integrity of cell walls to hinder pathogen invasion^62,63,69,70,106–110^. In addition, higher expression of spermine synthesis, directly following spermidine synthesis, has been shown to induce pathogen resistance^63,69^. NRS100 exhibits non-exclusive upregulation of spermine synthesis, downstream of exclusively upregulated SPDS. Therefore, accumulation of spermine and spermidine in response to SCN is likely. Conversely, it has been found that upregulation of SPDS can be induced by effector proteins secreted by nematodes, and therefore leads to a compatible plant–pathogen interaction^111^. Spermidine synthesis gene, SPDS17, is found to be significantly upregulated in NRS100, a response that is specific to the genotype. Interestingly, a separate SPDS gene on chromosome 15 is also highly upregulated in both Peking and NRS100, but not to the same extent as SPDS17 is in NRS100. In addition, upregulation of precursors, at putrescine synthesis (ODC1) is upregulated in both NRS100 and Peking but at a much higher level in NRS100. Overexpression of ODC1 has been shown to lead to elevated levels of putrescine but not spermidine, meaning that the upregulation of spermidine synthesis in NRS100 might activate an independent response and production of spermidine, regardless of the shared ODC1 upregulation with Peking^112,113^.

Although polyamine synthesis can be related to both compatible and incompatible interactions with pathogens, we propose that SPDS17-regulated spermidine synthesis in NRS100 might have a highly incompatible interaction with SCN^104^. The outcome impedes the nematodes’ ability to expand a feeding site, leading to decreased nutrition and death.

## Conclusion

These results indicated not only species-specific, but also genotype-specific mechanisms involved in SCN resistance. Here, we showed a distinct expression profile unique to a newly identified SCN-resistant wild soybean genotype, NRS100, in response to SCN and proposed candidate mechanisms associated with SCN resistance. The experiment design made use of both wild and cultivated soybeans to identify novel resistance mechanisms. The identification of candidate genes and pathways involved in SCN resistance advances the understanding of molecular mechanisms of plant and pest interactions. Prospectively, novel genes involved in SCN resistance can be incorporated into the cultivated soybean with diverse strategies, such as marker assisted breeding, gene transformation, genome editing technology, toward the long-term goal of developing new and diverse SCN resistant soybean cultivars, which has crucial significance to agriculture and environmental sustainability.

## Materials and methods

### Validation of NRS100 SCN resistance and experiment design

Our previous study showed high resistance of NRS100 to SCN race 5^44,45^. To verify this finding, we rescreened the NRS100 using race 5, along with indicator lines (Peking, PI88788, PI90763, PI437654, PI209332, PI89772, and PI548316), employed the same protocol described in Zhang et al.^44,45^. We also selected a susceptible wild soybean genotype, named S-*soja* (FI = 149)^44^, to compare the SCN-infection response differences with NRS100. Of the two main cultivated sources of resistance, Peking and PI88788, the former is the only one that is resistant to SCN race 5. Thus, we used Peking as a resistant soybean cultivar to test species-specific resistance strategies. We also used William82 as a susceptible soybean cultivar for comparison. All the study material were from USDA germplasm and this study comply with relevant institutional, national, and international guidelines.

### SCN hatching and stress treatment experiment

SCN race 5 were reared on soybean cv. Williams 82 plants in the greenhouse under optimal conditions at 27 °C and 15 h light for over 30 generations. Female SCN cysts were harvested from the roots using a nested sieve collection (850 and 250 µm) method, and females released from cysts by pressure into a 25 µm sieve^46^. Released eggs were purified by sucrose flotation^114^ and allowed to hatch on incubation trays over water. second stage juveniles (J2) were collected from the water and diluted to a concentration of 2500 eggs/ml for inoculation.

A controlled SCN stress experiment was performed on SCN-resistant wild soybean accession (NRS100), SCN-susceptible wild soybean accession (S-*soja*), SCN-resistant cultivated soybean accession (Peking), and SCN-susceptible cultivated soybean accession (Williams 82). Germinated soybeans were planted in cone planters (Stuewe & Sons, Tangent, Oregon, USA) in replicates of 12 per condition, treatment and control for each accession in sterilized sand with nutrients. After 2 days post transplanting, each plant was inoculated with hatched J2 of SCN race 5 (1500/plant) suspended in 0.09% agarose, or a blank (1 ml 0.09% agarose) for control groups. Optimal growing conditions were kept constant at 27 °C and 15 h light at 50% relative humidity in an environmental chamber. Roots were collected 6 days after SCN inoculation and fragmented in liquid nitrogen in preparation for RNA isolation. Each biological replicate contained pooled roots from three individual plants, providing four replicates of each group in total.

### Library construction and RNA sequencing

We used the same strategy and methods in root tissue collection, RNA extraction, RNA library preparation, and RNA sequencing as described in our pervious study^46,47^. Technical and biological replicates of total RNA sequences of controlled and treatment group root tissues were obtained using the Illumina 1.9 Hi-seq platform (NEB, Beverly, MA, USA). Each replicate was on two separate lanes and merged, generating 24 total libraries in triplicates per sample group (control 1–3, treatment 1–3 per genotype). Raw fastq reads were checked for quality control with FastQC (version 0.11.6) and filtered for quality, and trimmed (quality score < 30, and adapters) using FASTx toolkit (version 0.0.13). Reads were mapped against reference genome *G. max* Wm82.a2.v1^115^ with Tophat (version 2.1.1) and Bowtie2 (version 2.2.9) for all four genotypes, control and treatment. Assembly of transcripts of each replicate to the reference was done using Cufflinks (Version 2.2.1), a final assembly of each replicate was obtained, and differently expressed genes (DEGs) as the final step in the Cufflinks pipeline^116–119^.

### Comparative transcriptome analyses

In order to investigate the global gene expression changes, we compare RNA-seq-based transcriptomes of the NRS100, S-*soja*, Peking, and Williams 82, under both control and nematode-treated conditions. To determine induced response, differentially expressed profiles were found between control and treatment groups for each genotype. To determine constitutive expression, differentially expression profiles were compared between control conditions of each genotype.

Resulting transcripts from mapping, assembly by Tophat (version 2.1.1) and Bowtie2 (version 2.2.9) (were analyzed for differential expression using Cufflinks (Version 2.2.1))^118^. Genes with FDR significance (q < 0.01) were considered Differentially Expressed Genes DEGs. Putative metabolic pathways assigned for SCN-resistant genes with the KEGG pathway database, SoyBase Gene Model Data Mining and Analysis Tool (http://www.soybase.org)^120,121^, PlantRegMap^122^, and visualization of pathways in Pathview 1.22.3^123,124^. Integrative Genomics Viewer 2.5.3^125^ was used for sequence analysis against susceptible type Williams 82.

### Quantitative reverse transcription PCR (RT-qPCR)

Primers designed to bracket 80–150 bp of 20 selected genes (Table S6). RT-qPCR performed on an ABI 7500 Fast real-time PCR system (Applied Biosystems, Foster City, CA, USA) using PerfeCTa SYBR Green FastMix (Quanta Biosciences, Gaithersberg, MD, USA) with biological and technical triplicates. Relative expression quantified by comparison of ΔCT and FPKM from RNA sequencing results, in addition to the 2 − ΔΔCT method^126^.

## Supplementary Information

Supplementary Information 1.

Supplementary Information 2.

## Data Availability

The datasets generated during the current study are available in the NCBI Short Read Archive (SRA), Bioproject Accession: PRJNA664111, https://www.ncbi.nlm.nih.gov/bioproject/PRJNA664111/.

## References

[1] Wilson RF, Boerma HR, Specht JE (2004). Seed composition. Soybeans: Improvement, Production, and Uses. Agronomy Monograph.

[2] Wrather JA, Koenning SR (2006). Estimates of disease effects on soybean yields in the United States 2003 to 2005. J. Nematol..

[3] Tylka GL, Marett CC (2014). Distribution of the soybean cyst nematode, *Heterodera glycines*, in the United States and Canada: 1954 to 2014. Plant Health Progress..

[4] Mitchum MG (2016). Soybean resistance to the soybean cyst nematode *Heterodera glycines*: An update. Phytopathology.

[5] Kim M, Hyten DL, Niblack TL, Diers BW (2011). Stacking resistance alleles from wild and domestic soybean sources improves soybean cyst nematode resistance (vol 51, pg 934, 2011). Crop Sci..

[6] Wu X, Blake S, Sleper DA, Shannon JG, Cregan P, Nguyen HT (2009). QTL, additive and epistatic effects for SCN resistance in PI 437654. Theor. Appl. Genet..

[7] Gardner M, Heinz R, Wang J, Mitchum MG (2017). Genetics and adaptation of soybean cyst nematode to broad spectrum soybean resistance. Genes Genomes Genet..

[8] Niblack TL (2005). Soybean cyst nematode management reconsidered. Plant Dis..

[9] Niblack T, Colgrove A, Colgrove K, Bond J (2008). Shift in virulence of soybean cyst nematode is associated with use of resistance from PI 88788. Plant Health Progress.

[10] Caldwell BE, Brim CA, Ross JP (1960). Inheritance of resistance of soybeans to the cyst nematode, *Heterodera glycines*1. Agron. J..

[11] Hauge BM, Wang ML, Parsons JD, Parnell LD (2006). Nucleic acid molecules and other molecules associated with soybean cyst nematode resistance. Google Patents.

[12] Lightfoot D, Meksem K (2002). Isolated polynucleotides and polypeptides relating to loci underlying resistance to soybean cyst nematode and soybean sudden death syndrome and methods employing same. Google Patents.

[13] Liu X, Liu S, Jamai A, Bendahmane A, Lightfoot DA, Mitchum MG (2011). Soybean cyst nematode resistance in soybean is independent of the Rhg4 locus LRR-RLK gene. Funct. Integr. Genom..

[14] Melito S, Heuberger AL, Cook D, Diers BW, MacGuidwin AE, Bent AF (2010). A nematode demographics assay in transgenic roots reveals no significant impacts of the Rhg1locus LRR-Kinase on soybean cyst nematode resistance. BMC Plant Biol..

[15] Cook DE, Bayless AM, Wang K, Guo X, Song Q, Jiang J (2014). Distinct copy number, coding sequence, and locus methylation patterns underlie *Rhg1*-mediated soybean resistance to soybean cyst nematode. Plant Physiol..

[16] Cook DE, Lee TG, Guo XL, Melito S, Wang K, Bayless AM (2012). Copy number variation of multiple genes at Rhg1 mediates nematode resistance in soybean. Science.

[17] Liu S, Kandoth PK, Warren SD, Yeckel G, Heinz R, Alden J (2012). A soybean cyst nematode resistance gene points to a new mechanism of plant resistance to pathogens. Nature.

[18] Liu SM, Kandoth PK, Lakhssassi N, Kang JW, Colantonio V, Heinz R (2017). The soybean GmSNAP18 gene underlies two types of resistance to soybean cyst nematode. Nat. Commun..

[19] Yu N, Lee TG, Rosa DP, Hudson M, Diers BW (2016). Impact of Rhg1 copy number, type, and interaction with Rhg4 on resistance to *Heterodera glycines* in soybean. Theor. Appl. Genet..

[20] Patil GB, Lakhssassi N, Wan J, Song L, Zhou Z, Klepadlo M (2019). Whole-genome re-sequencing reveals the impact of the interaction of copy number variants of the rhg1 and Rhg4 genes on broad-based resistance to soybean cyst nematode. Plant Biotechnol. J...

[21] Meksem K, Liu S, Pramod K, Lakhssassi N, Colantonio V, Kang JW (2017). The GmSNAP18 is the Peking-type rhg1-a gene for resistance to soybean cyst nematode. Phytopathology.

[22] Lee TG, Kumar I, Diers BW, Hudson ME (2015). Evolution and selection of Rhg1, a copy-number variant nematode-resistance locus. Mol. Ecol..

[23] Wu X-Y, Zhou G-C, Chen Y-X, Wu P, Liu L-W, Ma F-F (2016). Soybean cyst nematode resistance emerged via artificial selection of duplicated serine hydroxymethyltransferase genes. Front. Plant Sci..

[24] Hyten DL, Song Q, Zhu Y, Choi I-Y, Nelson RL, Costa JM (2006). Impacts of genetic bottlenecks on soybean genome diversity. Proc. Natl. Acad. Sci..

[25] Zhou Z, Jiang Y, Wang Z, Gou Z, Lyu J, Li W (2015). Resequencing 302 wild and cultivated accessions identifies genes related to domestication and improvement in soybean. Nat. Biotechnol..

[26] Wen ZX, Boyse JF, Song QJ, Cregan PB, Wang DC (2015). Genomic consequences of selection and genome-wide association mapping in soybean. BMC Genom..

[27] Peng D, Peng H, Wu D, Huang W, Ye W, Cui J (2016). First report of soybean cyst nematode (*Heterodera glycines*) on soybean from Gansu and Ningxia China. Plant Dis..

[28] Yan GP, Baidoo R (2018). Current research status of *Heterodera glycines* resistance and its implication on soybean breeding. Eng. Proc..

[29] He SL, Wang YS, Li DZ, Yi TS (2016). Environmental and historical determinants of patterns of genetic differentiation in wild soybean (Glycine soja Sieb. et Zucc). Sci. Rep..

[30] Kofsky J, Zhang HY, Song BH (2018). The untapped genetic reservoir: The past, current, and future applications of the wild soybean (Glycine soja). Front Plant Sci..

[31] Ning W, Zhai H, Yu J, Liang S, Yang X, Xing X (2017). Overexpression of Glycine soja WRKY20 enhances drought tolerance and improves plant yields under drought stress in transgenic soybean. Mol. Breed..

[32] Munns R, James RA, Xu B, Athman A, Conn SJ, Jordans C (2012). Wheat grain yield on saline soils is improved by an ancestral Na+ transporter gene. Nat. Biotechnol..

[33] Arbelaez JD, Moreno LT, Singh N, Tung C-W, Maron LG, Ospina Y (2015). Development and GBS-genotyping of introgression lines (ILs) using two wild species of rice, *O. meridionalis* and *O. rufipogon*, in a common recurrent parent, *O. sativa* cv. Curinga. Mol. Breed..

[34] Von Korff M, Wang H, Léon J, Pillen K (2004). Development of candidate introgression lines using an exotic barley accession (*Hordeum vulgare* ssp. spontaneum) as donor. Theoret. Appl. Genet..

[35] Naz AA, Arifuzzaman M, Muzammil S, Pillen K, Léon J (2014). Wild barley introgression lines revealed novel QTL alleles for root and related shoot traits in the cultivated barley (*Hordeum vulgare* L.). BMC Genet..

[36] Hajjar R, Hodgkin T (2007). The use of wild relatives in crop improvement: A survey of developments over the last 20 years. Euphytica.

[37] Zhou Y-L, Xu J-L, Zhou S-C, Yu J, Xie X-W, Xu M-R (2009). Pyramiding Xa23 and Rxo1 for resistance to two bacterial diseases into an elite indica rice variety using molecular approaches. Mol. Breed..

[38] Ballini E, Berruyer R, Morel JB, Lebrun MH, Nottéghem JL, Tharreau D (2007). Modern elite rice varieties of the ‘Green Revolution’have retained a large introgression from wild rice around the Pi33 rice blast resistance locus. New Phytol..

[39] Menda N, Strickler SR, Edwards JD, Bombarely A, Dunham DM, Martin GB (2014). Analysis of wild-species introgressions in tomato inbreds uncovers ancestral origins. BMC Plant Biol..

[40] Kumar A, Tiwari K, Datta D, Singh M (2014). Marker assisted gene pyramiding for enhanced Tomato leaf curl virus disease resistance in tomato cultivars. Biol. Plant..

[41] Stupar RM (2010). Into the wild: The soybean genome meets its undomesticated relative. Proc. Natl. Acad. Sci..

[42] Carter TE, Hymowitz T, Nelson RL (2004). Biogeography, local adaptation, vavilov, and genetic diversity in soybean. Biol. Resour. Migrat..

[43] Zhang H, Mittal N, Leamy LJ, Barazani O, Song BH (2017). Back into the wild—apply untapped genetic diversity of wild relatives for crop improvement. Evol. Appl..

[44] Zhang H, Li C, Davis EL, Wang J, Griffin JD, Kofsky J (2016). Genome-wide association study of resistance to soybean cyst nematode (*Heterodera glycines*) HG Type 2.5.7 in wild soybean (Glycine soja). Front Plant Sci..

[45] Zhang HY, Song QJ, Griffin JD, Song BH (2017). Genetic architecture of wild soybean (*Glycine soja*) response to soybean cyst nematode (*Heterodera glycines*). Mol. Genet. Genom..

[46] Zhang H, Kjemtrup-Lovelace S, Li C, Luo Y, Chen LP, Song BH (2017). Comparative RNA-seq analysis uncovers a complex regulatory network for soybean cyst nematode resistance in wild soybean (*Glycine soja*). Sci. Rep..

[47] Zhang HY, Song BH (2017). RNA-seq data comparisons of wild soybean genotypes in response to soybean cyst nematode (*Heterodera glycines*). Genom Data.

[48] Concibido VC, Diers BW, Arelli PR (2004). A decade of QTL mapping for cyst nematode resistance in soybean. Crop Sci..

[49] Meksem K, Pantazopoulos P, Njiti VN, Hyten LD, Arelli PR, Lightfoot DA (2001). ’Forrest’ resistance to the soybean cyst nematode is bigenic: Saturation mapping of the Rhg1and Rhg4 loci. Theor. Appl. Genet..

[50] Lakhssassi N, Liu S, Bekal S, Zhou Z, Colantonio V, Lambert K (2017). Characterization of the soluble NSF attachment protein gene family identifies two members involved in additive resistance to a plant pathogen. Sci. Rep..

[51] Kim M, Diers BW (2013). Fine mapping of the SCN resistance QTL cqSCN-006 and cqSCN-007 from Glycine soja PI 468916. Crop Sci..

[52] Wang D, Diers BW, Arelli PR, Shoemaker RC (2001). Loci underlying resistance to Race 3 of soybean cyst nematode in Glycine soja plant introduction 468916. Theor. Appl. Genet..

[53] Yu N, Diers BW (2017). Fine mapping of the SCN resistance QTL cqSCN-006 and cqSCN-007 from Glycine soja PI 468916. Euphytica.

[54] Zhou L, Song L, Lian Y, Ye H, Usovsky M, Wan J (2021). Genetic characterization of qSCN10 from an exotic soybean accession PI 567516C reveals a novel source conferring broad-spectrum resistance to soybean cyst nematode. Theoret. Appl. Genet..

[55] Riechmann JL, Meyerowitz EM (1998). The AP2/EREBP family of plant transcription factors. Biol. Chem..

[56] Oliveros JC. VENNY. An interactive tool for comparing lists with Venn Diagrams. http://bioinfogp.cnb.csic.es/tools/venny/index.html. 2007.

[57] Dixon RA (2001). Natural products and plant disease resistance. Nature.

[58] Durner J, Shah J, Klessig DF (1997). Salicylic acid and disease resistance in plants. Trends Plant Sci..

[59] Lang J, Genot B, Hirt H, Colcombet J (2017). Constitutive activity of the Arabidopsis MAP Kinase 3 confers resistance to *Pseudomonas syringae* and drives robust immune responses. Plant Signal. Behav..

[60] Wittstock U, Gershenzon J (2002). Constitutive plant toxins and their role in defense against herbivores and pathogens. Curr. Opin. Plant Biol..

[61] Zhang J, Peng Y, Guo Z (2007). Constitutive expression of pathogen-inducible OsWRKY31 enhances disease resistance and affects root growth and auxin response in transgenic rice plants. Cell Res..

[62] Angelini R, Bragaloni M, Federico R, Infantino A, Porta-Pugua A (1993). Involvement of polyamines, diamine oxidase and peroxidase in resistance of chickpea to *Ascochyta rabiei*. J. Plant Physiol..

[63] Yoda H, Fujimura K, Takahashi H, Munemura I, Uchimiya H, Sano H (2009). Polyamines as a common source of hydrogen peroxide in host- and nonhost hypersensitive response during pathogen infection. Plant Mol. Biol..

[64] Ali MA, Anjam MS, Nawaz MA, Lam HM, Chung G (2018). Signal transduction in plant–nematode interactions. Int. J. Mol. Sci..

[65] McConn M, Creelman RA, Bell E, Mullet JE, Browse J (1997). Jasmonate is essential for insect defense Arabidopsis. Proc. Natl. Acad. Sci. USA.

[66] Nahar K, Kyndt T, De Vleesschauwer D, Hofte M, Gheysen G (2011). The jasmonate pathway is a key player in systemically induced defense against root knot nematodes in rice. Plant Physiol..

[67] Zhang L, Zhang F, Melotto M, Yao J, He SY (2017). Jasmonate signaling and manipulation by pathogens and insects. J. Exp. Bot..

[68] Reymond P, Farmer EE (1998). Jasmonate and salicylate as global signals for defense gene expression. Curr. Opin. Plant Biol..

[69] Takahashi Y (2016). The role of polyamines in plant disease resistance. Environ. Control. Biol..

[70] Walters D (2003). Resistance to plant pathogens: Possible roles for free polyamines and polyamine catabolism. New Phytol..

[71] Howland A, Monnig N, Mathesius J, Nathan M, Mitchum MG (2018). Survey of *Heterodera glycines* population densities and virulence phenotypes during 2015–2016 in Missouri. Plant Dis..

[72] Bayless AM, Zapotocny RW, Grunwald DJ, Amundson KK, Diers BW, Bent AF (2018). An atypical *N*-ethylmaleimide sensitive factor enables the viability of nematode-resistant Rhg1 soybeans. Proc. Natl. Acad. Sci..

[73] Kabelka EA, Carlson SR, Diers BW (2005). Localization of two loci that confer resistance to soybean cyst nematode from Glycine soja PI 468916. Crop Sci..

[74] Yuan C, Zhang L, Zhao H, Wang Y, Liu X, Dong Y (2019). RNA-seq analysis for soybean cyst nematode resistance of *Glycine soja* (wild soybean). Oil Crop Sci..

[75] Bayless AM, Smith JM, Song J, McMinn PH, Teillet A, August BK (2016). Disease resistance through impairment of α-SNAP–NSF interaction and vesicular trafficking by soybean Rhg1. Proc. Natl. Acad. Sci..

[76] Bekal S, Domier LL, Gonfa B, Lakhssassi N, Meksem K, Lambert KN (2015). A SNARE-like protein and biotin are implicated in soybean cyst nematode virulence. PLoS One.

[77] Zander M, Thurow C, Gatz C (2014). TGA transcription factors activate the salicylic acid-suppressible branch of the ethylene-induced defense program by regulating ORA59 expression. Plant Physiol..

[78] Chang KN, Zhong S, Weirauch MT, Hon G, Pelizzola M, Li H (2013). Temporal transcriptional response to ethylene gas drives growth hormone cross-regulation in Arabidopsis. Elife.

[79] McGrath KC, Dombrecht B, Manners JM, Schenk PM, Edgar CI, Maclean DJ (2005). Repressor-and activator-type ethylene response factors functioning in jasmonate signaling and disease resistance identified via a genome-wide screen of Arabidopsis transcription factor gene expression. Plant Physiol..

[80] Nakano T, Suzuki K, Ohtsuki N, Tsujimoto Y, Fujimura T, Shinshi H (2006). Identification of genes of the plant-specific transcription-factor families cooperatively regulated by ethylene and jasmonate in *Arabidopsis thaliana*. J. Plant. Res..

[81] Hu Y, You J, Li C, Williamson VM, Wang C. Ethylene response pathway modulates attractiveness of plant roots to soybean cyst nematode *Heterodera glycines*. Scientific reports. 2017;7:41282-. 10.1038/srep41282 PMC5256374 28112257

[82] Klink VP, Hosseini P, Matsye PD, Alkharouf NW, Matthews BF (2010). Syncytium gene expression in *Glycine max* [PI 88788] roots undergoing a resistant reaction to the parasitic nematode *Heterodera glycines*. Plant Physiol. Biochem..

[83] Li R, Rashotte AM, Singh NK, Weaver DB, Lawrence KS, Locy RD (2015). Integrated signaling networks in plant responses to sedentary endoparasitic nematodes: A perspective. Plant Cell Rep..

[84] Mazarei M, Puthoff DP, Hart JK, Rodermel SR, Baum TJ (2002). Identification and characterization of a soybean ethylene-responsive element-binding protein gene whose mRNA expression changes during soybean cyst nematode infection. Mol. Plant Microbe Interact..

[85] Nahar K, Kyndt T, De Vleesschauwer D, Höfte M, Gheysen G (2011). The jasmonate pathway is a key player in systemically induced defense against root knot nematodes in rice. Plant Physiol..

[86] Tucker ML, Xue P, Yang R (2009). 1-Aminocyclopropane-1-carboxylic acid (ACC) concentration and ACC synthase expression in soybean roots, root tips, and soybean cyst nematode (*Heterodera glycines*)-infected roots. J. Exp. Bot..

[87] Feys BJ, Parker JE (2000). Interplay of signaling pathways in plant disease resistance. Trends Genet..

[88] Farmer EE, Ryan CA (1992). Octadecanoid precursors of jasmonic acid activate the synthesis of wound-inducible proteinase inhibitors. Plant Cell.

[89] Ruan J, Zhou Y, Zhou M, Yan J, Khurshid M, Weng W (2019). Jasmonic acid signaling pathway in plants. Int. J. Mol. Sci..

[90] Okada K, Abe H, Arimura G-I (2014). Jasmonates induce both defense responses and communication in monocotyledonous and dicotyledonous plants. Plant Cell Physiol..

[91] Avila CA, Arévalo-Soliz LM, Jia L, Navarre DA, Chen Z, Howe GA (2012). Loss of function of FATTY ACID DESATURASE7 in tomato enhances basal aphid resistance in a salicylate-dependent manner. Plant Physiol..

[92] Beneventi MA, da Silva OB, de Sá MEL, Firmino AAP, de Amorim RMS, Albuquerque ÉVS (2013). Transcription profile of soybean-root-knot nematode interaction reveals a key role of phythormones in the resistance reaction. BMC Genom..

[93] Zhou G, Qi J, Ren N, Cheng J, Erb M, Mao B (2009). Silencing OsHI-LOX makes rice more susceptible to chewing herbivores, but enhances resistance to a phloem feeder. Plant J..

[94] Felton GW, Korth KL, Bi JL, Wesley SV, Huhman DV, Mathews MC (1999). Inverse relationship between systemic resistance of plants to microorganisms and to insect herbivory. Curr. Biol..

[95] Gupta V, Willits MG, Glazebrook J (2000). Arabidopsis thaliana EDS4 contributes to salicylic acid (SA)-dependent expression of defense responses: Evidence for inhibition of jasmonic acid signaling by SA. Mol. Plant Microbe Interact..

[96] Turner JG, Ellis C, Devoto A (2002). The jasmonate signal pathway. Plant Cell.

[97] Yang D-L, Yao J, Mei C-S, Tong X-H, Zeng L-J, Li Q (2012). Plant hormone jasmonate prioritizes defense over growth by interfering with gibberellin signaling cascade. Proc. Natl. Acad. Sci..

[98] Musser RO, Hum-Musser SM, Eichenseer H, Peiffer M, Ervin G, Murphy JB (2002). Herbivory: Caterpillar saliva beats plant defences. Nature.

[99] Kus JV, Zaton K, Sarkar R, Cameron RK (2002). Age-related resistance in Arabidopsis is a developmentally regulated defense response to *Pseudomonas syringae*. Plant Cell.

[100] Zhai Q, Zhang X, Wu F, Feng H, Deng L, Xu L (2015). Transcriptional mechanism of jasmonate receptor COI1-mediated delay of flowering time in Arabidopsis. Plant Cell.

[101] Xie D-X, Feys BF, James S, Nieto-Rostro M, Turner JG (1998). COI1: An Arabidopsis gene required for jasmonate-regulated defense and fertility. Science.

[102] Thines B, Katsir L, Melotto M, Niu Y, Mandaokar A, Liu G (2007). JAZ repressor proteins are targets of the SCFCOI1 complex during jasmonate signalling. Nature.

[103] Chini A, Fonseca S, Fernández G, Adie B, Chico JM, Lorenzo O (2007). The JAZ family of repressors is the missing link in jasmonate signalling. Nature.

[104] Walters DR (2003). Polyamines and plant disease. Phytochemistry.

[105] Walters DR (2000). Polyamines in plant–microbe interactions. Physiol. Mol. Plant Pathol..

[106] Fu X-Z, Chen C-W, Wang Y, Liu J-H, Moriguchi T (2011). Ectopic expression of MdSPDS1 in sweet orange (Citrus sinensis Osbeck) reduces canker susceptibility: Involvement of H_2_O_2_ production and transcriptional alteration. BMC Plant Biol..

[107] Seifi HS, Shelp BJ (2019). Spermine differentially refines plant defense responses against biotic and abiotic stresses. Front Plant Sci..

[108] Pal M, Janda T (2017). Role of polyamine metabolism in plant pathogen interactions. J. Plant Sci. Phytopathol..

[109] Berta G, Altamura MM, Fusconi A, Cerruti F, Capitani F, Bagni N (1997). The plant cell wall is altered by inhibition of polyamine biosynthesis. New Phytol..

[110] Martin-Tanguy J (1997). Conjugated polyamines and reproductive development: Biochemical, molecular and physiological approaches. Physiol. Plant..

[111] Hewezi T, Howe PJ, Maier TR, Hussey RS, Mitchum MG, Davis EL (2010). Arabidopsis spermidine synthase is targeted by an effector protein of the Cyst nematode *Heterodera schachtii*. Plant Physiol..

[112] Capell T, Escobar C, Liu H, Burtin D, Lepri O, Christou P (1998). Over-expression of the oat arginine decarboxylase cDNA in transgenic rice (*Oryza sativa* L.) affects normal development patterns in vitro and results in putrescine accumulation in transgenic plants. Theoret. Appl. Genet..

[113] Bhatnagar P, Glasheen BM, Bains SK, Long SL, Minocha R, Walter C (2001). Transgenic manipulation of the metabolism of polyamines in poplar cells. Plant Physiol..

[114] Matthews B, MacDonald M, Thai V, Tucker M (2003). Molecular characterization of arginine kinases in the soybean cyst nematode (*Heterodera glycines*). J. Nematol..

[115] Schmutz J, Cannon SB, Schlueter J, Ma J, Mitros T, Nelson W (2010). Genome sequence of the palaeopolyploid soybean. Nature.

[116] Roberts A, Pimentel H, Trapnell C, Pachter L (2011). Identification of novel transcripts in annotated genomes using RNA-Seq. Bioinformatics.

[117] Roberts A, Trapnell C, Donaghey J, Rinn JL, Pachter L (2011). Improving RNA-Seq expression estimates by correcting for fragment bias. Genome Biol..

[118] Trapnell C, Hendrickson DG, Sauvageau M, Goff L, Rinn JL, Pachter L (2012). Differential analysis of gene regulation at transcript resolution with RNA-seq. Nat. Biotechnol..

[119] Trapnell C, Williams BA, Pertea G, Mortazavi A, Kwan G, van Baren MJ (2010). Transcript assembly and quantification by RNA-Seq reveals unannotated transcripts and isoform switching during cell differentiation. Nat. Biotechnol..

[120] Morales AMAP, O'Rourke JA, van de Mortel M, Scheider KT, Bancroft TJ, Borém A (2013). Transcriptome analyses and virus induced gene silencing identify genes in the *Rpp4*-mediated Asian soybean rust resistance pathway. Funct. Plant Biol..

[121] Grant D, Nelson RT, Cannon SB, Shoemaker RC (2009). SoyBase, the USDA-ARS soybean genetics and genomics database. Nucleic Acids Res..

[122] Jin J, He K, Tang X, Li Z, Lv L, Zhao Y (2015). An Arabidopsis transcriptional regulatory map reveals distinct functional and evolutionary features of novel transcription factors. Mol. Biol. Evol..

[123] Luo W, Brouwer C (2013). Pathview: An R/bioconductor package for pathway-based data integration and visualization. Bioinformatics.

[124] Luo W, Pant G, Bhavnasi YK, Blanchard SG, Brouwer C (2017). Pathview Web: User friendly pathway visualization and data integration. Nucleic Acids Res..

[125] Robinson JT, Thorvaldsdóttir H, Winckler W, Guttman M, Lander ES, Getz G (2011). Integrative genomics viewer. Nat. Biotechnol..

[126] Livak KJ, Schmittgen TD (2001). Analysis of relative gene expression data using real-time quantitative PCR and the 2− ΔΔCT method. Methods.

