# Supplementary material for: Novel resistance strategies to soybean cyst nematode (SCN) in wild soybean
Source: Sci Rep. 2021 Apr 12;11:7967. doi: 10.1038/s41598-021-86793-z (PMC8041904; doi:10.1038/s41598-021-86793-z)

Full Title: Novel resistance strategies to soybean cyst nematode (SCN) in wild soybean

Short Title: Novel SCN resistance in wild soybean

Authors: Janice Kofsky, Hengyou Zhang[#], and Bao-Hua Song[*]

Affiliation: Department of Biological Sciences, University of North Carolina at Charlotte, Charlotte, NC 28223, USA

***Corresponding author**:

Bao-Hua Song (bsong5@uncc.edu)

[#]: Current Affiliation: Donald Danforth Plant Science Center, Saint Louis, MO 63132, USA

Fig. S1. Graph of all accessions screened for SCN resistance with resulting FI%. NRS100 is the most resistant of all screened accessions.

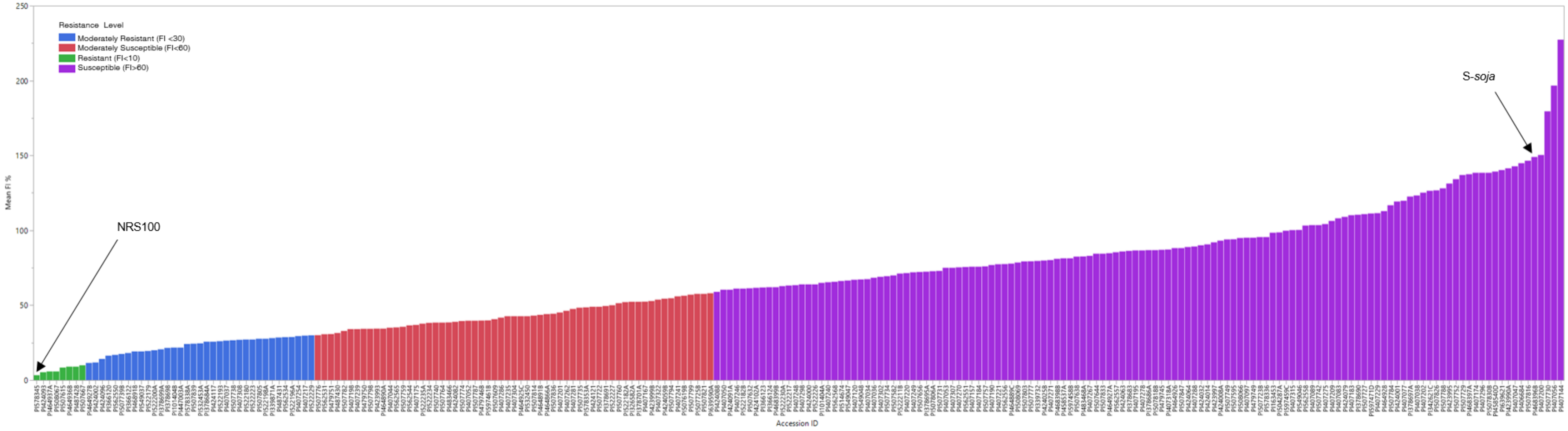

Fig. S2. *Rhg1* gene *SNAP (Glyma.18G022500)* comparison of nucleotide sequence and amino acid sequence between Peking, Williams 82, S-*soja*, and NRS100. Variations are highlighted in gray. Synonymous variations are outlined in gray. Non-synonymous variations are outlined in red.

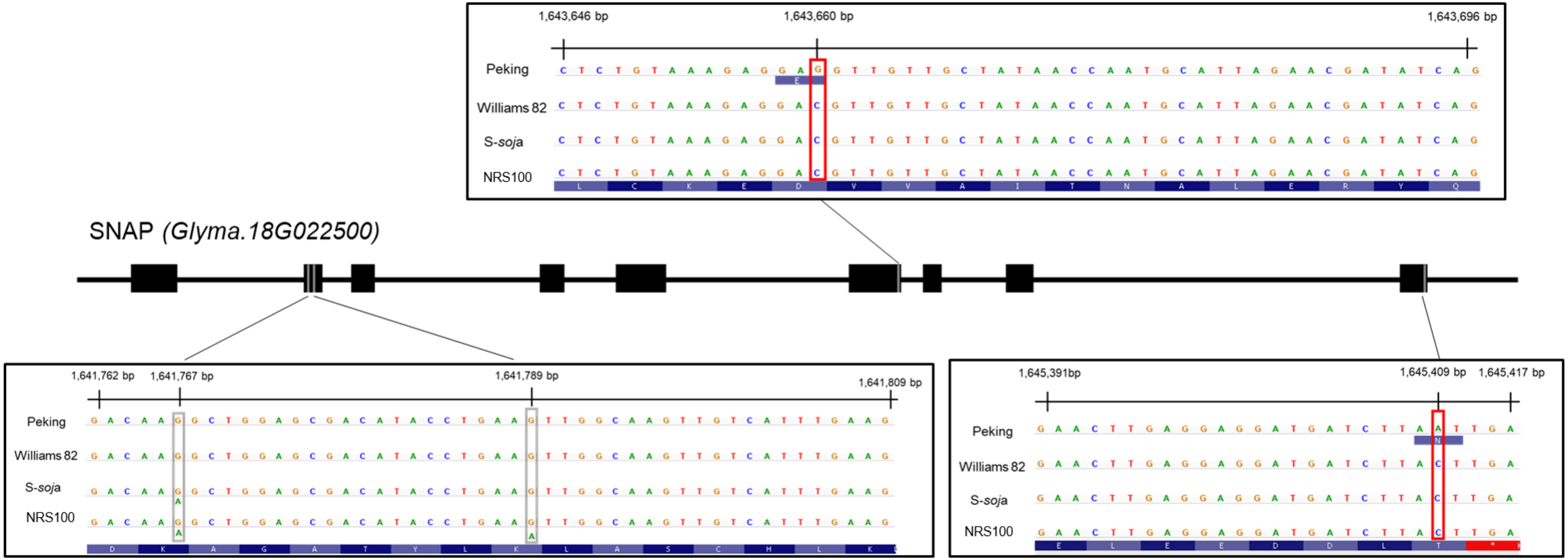

Fig. S3. *Rhg4* gene *SHMT (Glyma.08G108900)* comparison of nucleotide sequence and amino acid sequence between Peking, Williams 82, S-*soja*, and NRS100. Variations are highlighted in gray. Synonymous variations are outlined in gray. Non-synonymous variations are outlined in red.

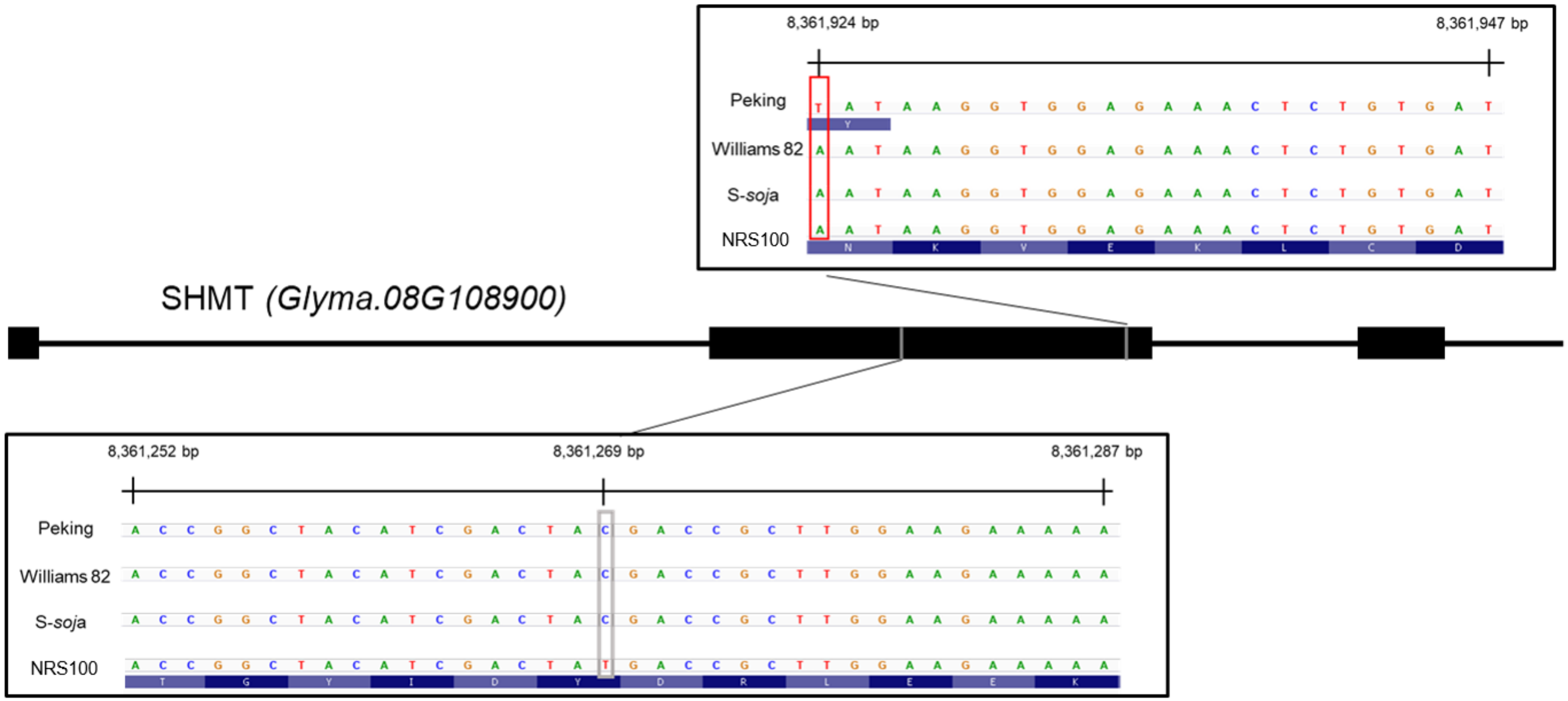

Fig. S4. Relative fold change of grouped replicates calculated by $2^{-\Delta\Delta CT}$ method of SNAP genes. No significant fold change found.

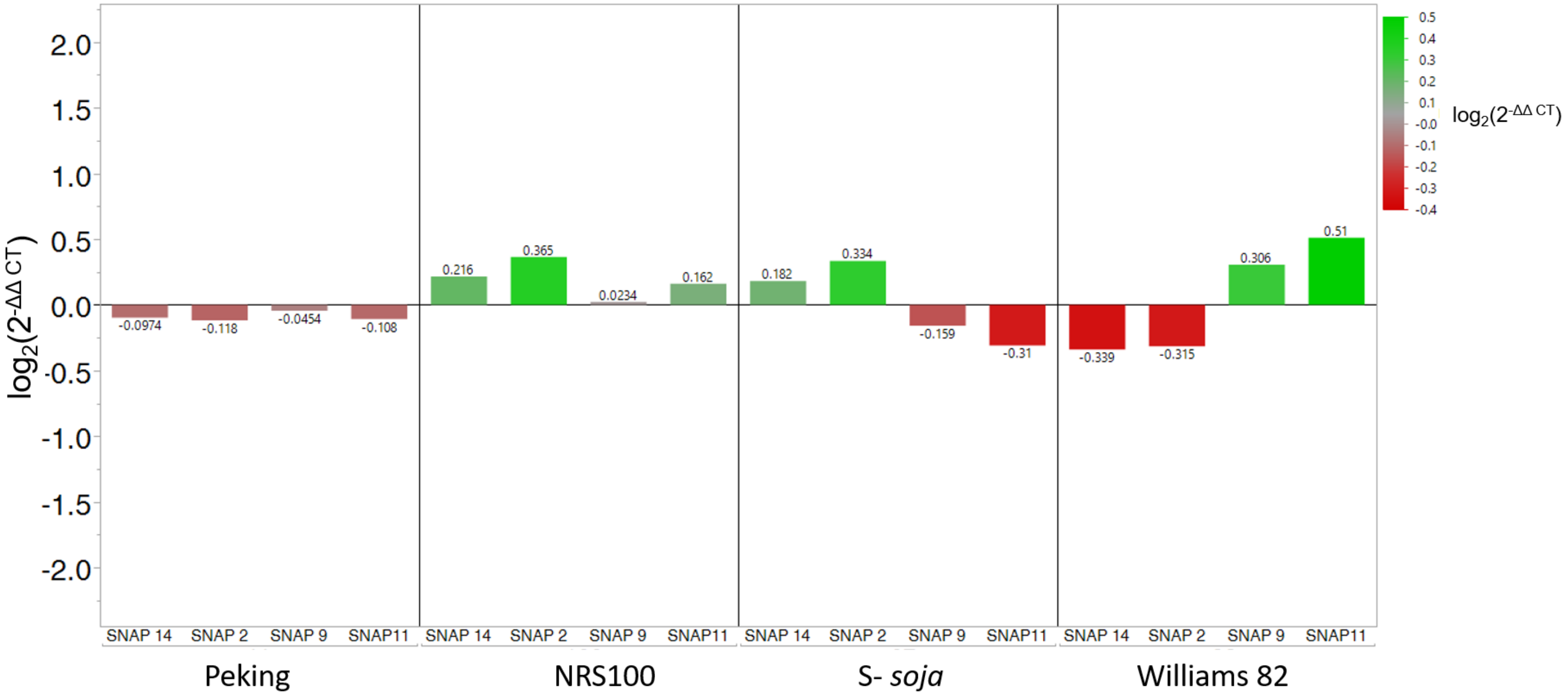

Fig. S5. Overrepresented GO functions for NRS100 and Peking resistance specific genes. Significance cut-off at bonferroni adjusted p-value <0.01.

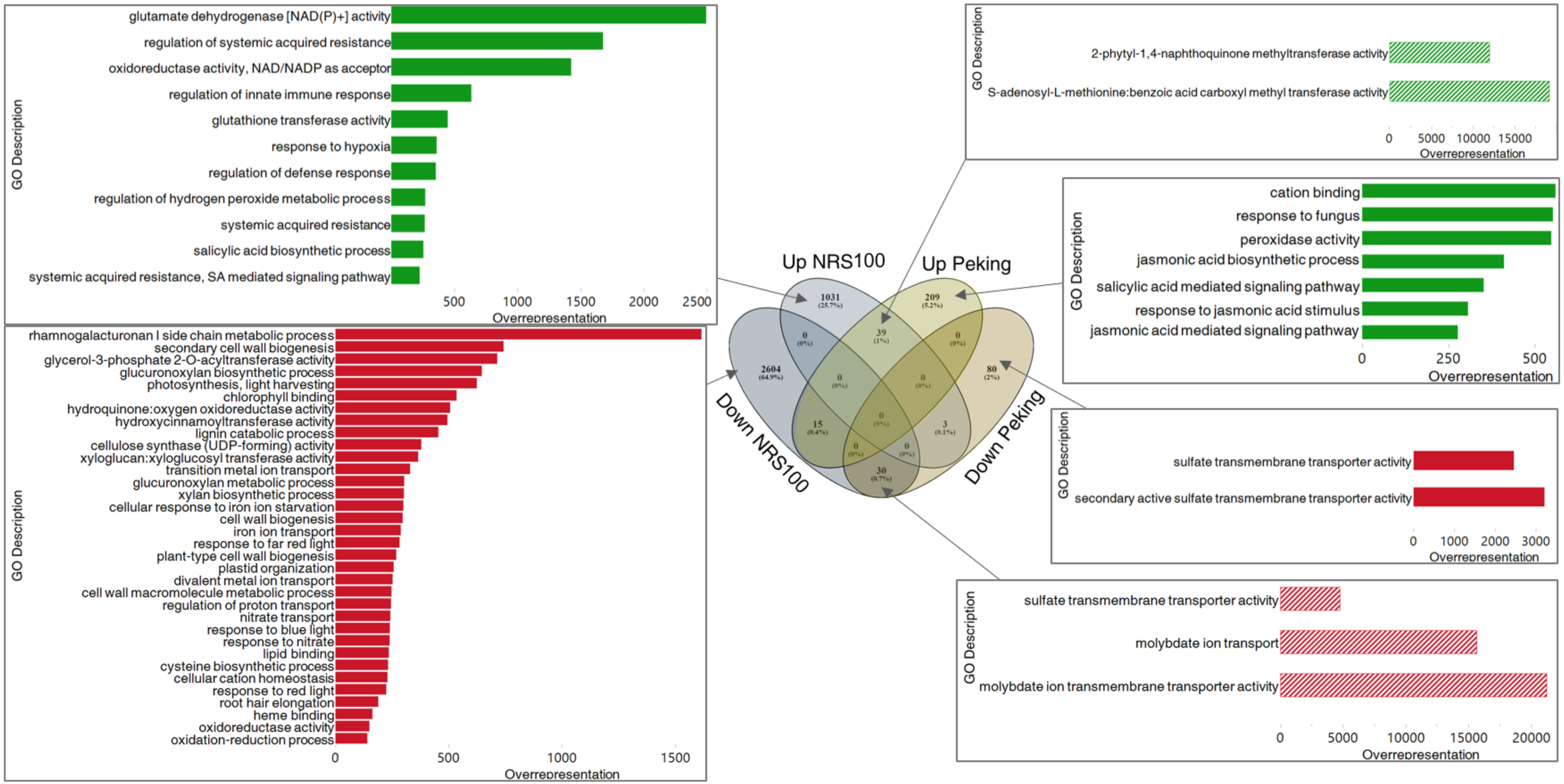

Fig. S6. Significantly highly expressed genes in NRS100 control expression profile (NRS100 (C)), compared to S-*soja* control expression profile (S-soja (C)) and to Peking control expression profile (Peking (C)), q-value<0.01. NRS100 SCN induced expression profiles, both up (NRS100 (T) Up) and down (NRS100 (T) Down), compared to the NRS100-specific control expressed genes.

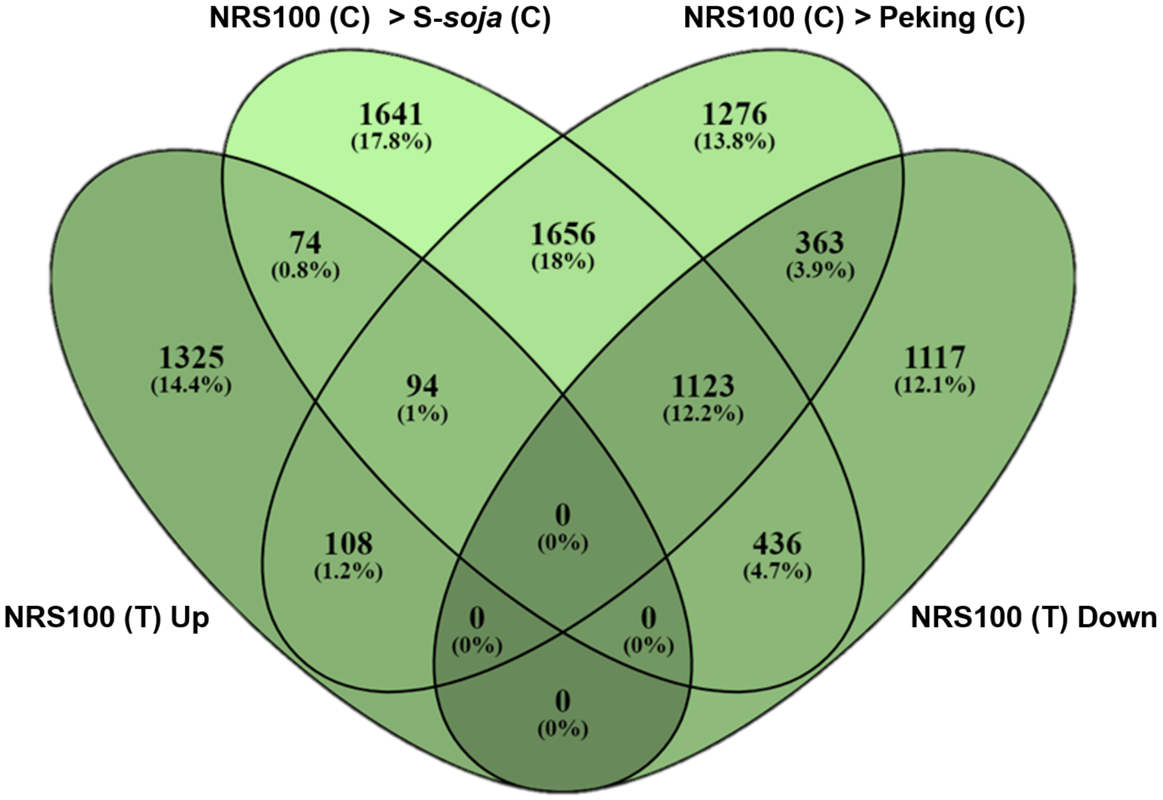

Fig. S7. Significantly enriched GO functions (p<0.01) of the 1656 NRS100-specific significantly constitutively expressed genes.

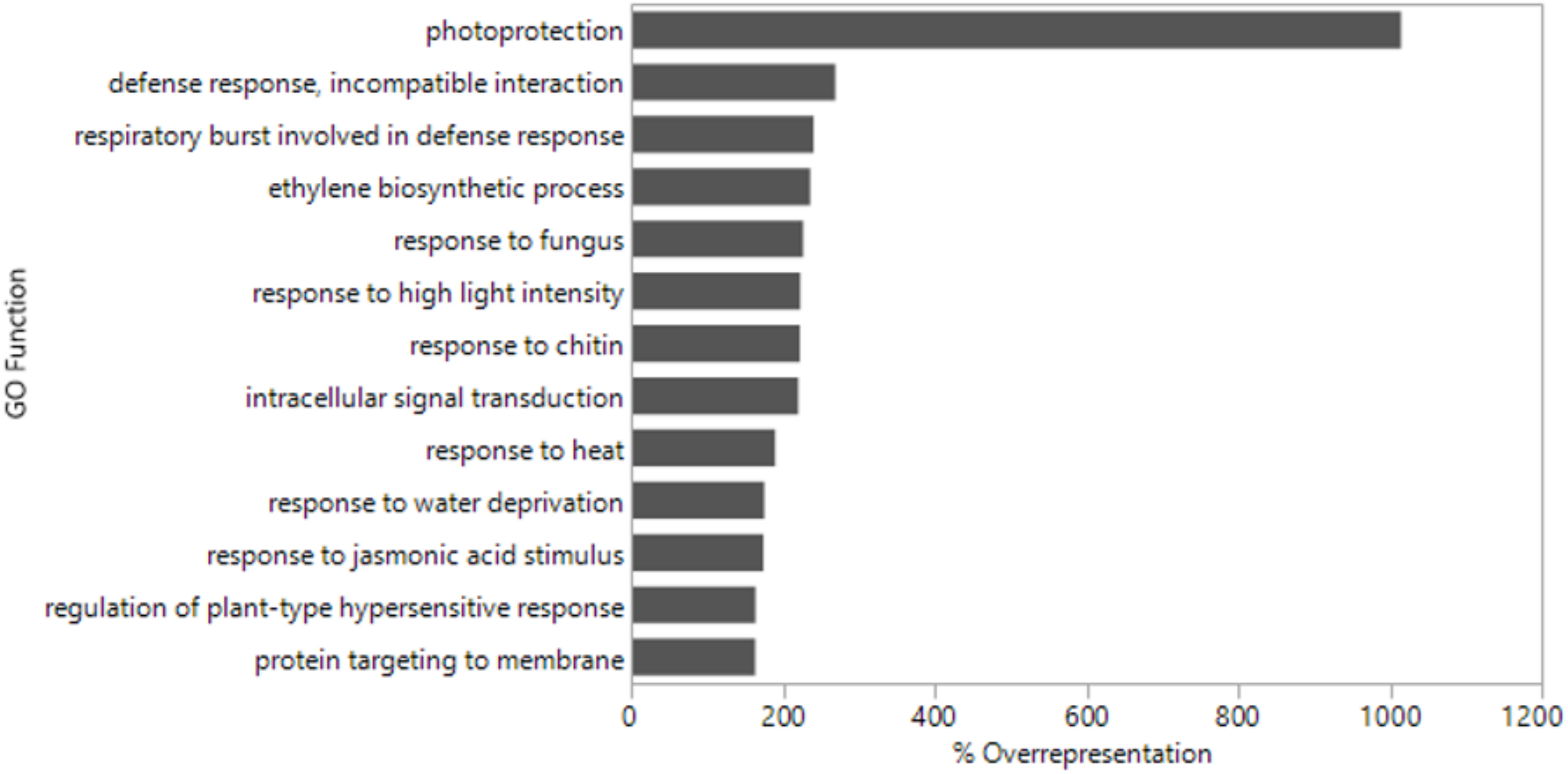

Fig. S8. Heatmap/Pathway of DEGs by log_2FC (fold change) of constitute expression relative to other genotypes on left of box and induced response to SCN treatment on right of box. NRS100 specific regulation is shown with purple stars. Adapted from PATHVIEW output.

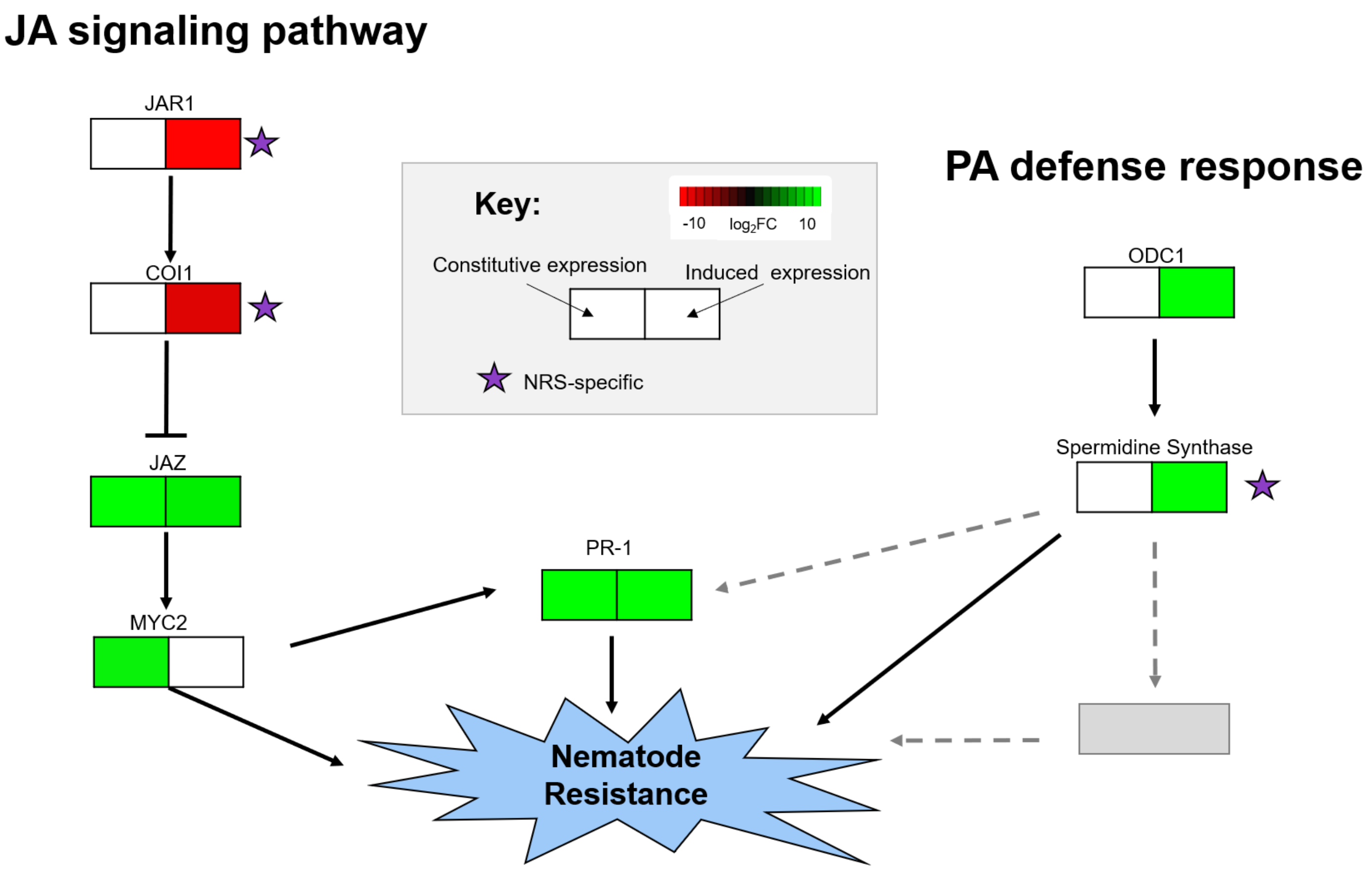

Fig. S9. Positive correlation (R^2=0.752) between qPCR fold change expression values ($log_2(2^{-\Delta\Delta CT})$) and correlated RNA-sequencing fold change expression values (log_2(FC)) where FC= Fold change, for 20 genes (Table S6). Individual points represent relative fold change of grouped replicates calculated by $2^{-\Delta\Delta CT}$ method and calculated fold change of group replicates from RNA-sequencing analysis.

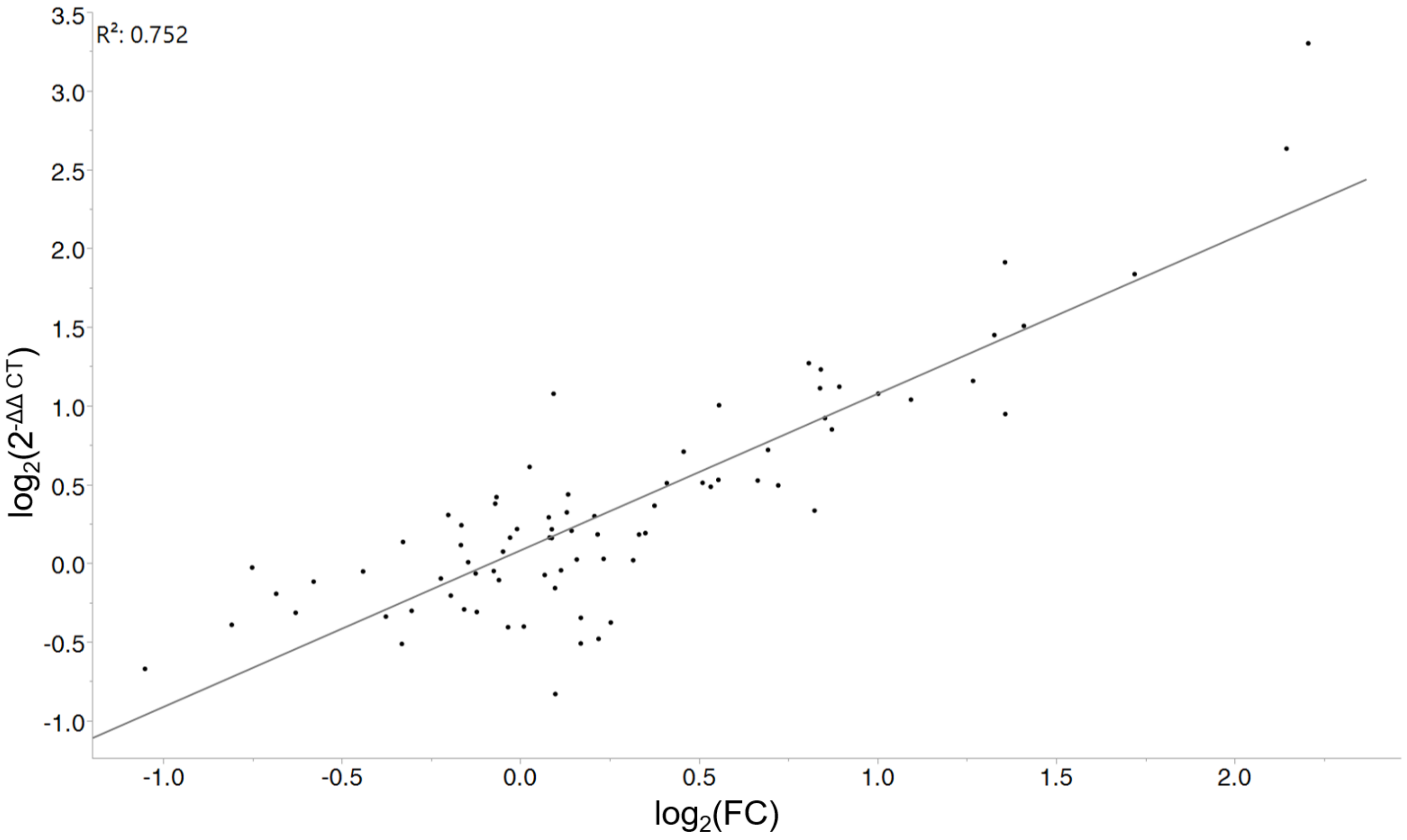

Supplement: Supplementary file 1 — Supplementary Information 1. [file 41598_2021_86793_MOESM1_ESM.pdf]
